# KDM6B promotes gastric carcinogenesis and metastasis via upregulation of CXCR4 expression

**DOI:** 10.1038/s41419-022-05458-5

**Published:** 2022-12-23

**Authors:** Fen Liu, Yue Wang, Zongcheng Yang, Xiujie Cui, Lixin Zheng, Yue Fu, Wei Shao, Lu Zhang, Qing Yang, Jihui Jia

**Affiliations:** 1 Department of Microbiology/Key Laboratory for Experimental Teratology of the Chinese Ministry of Education, School of Basic Medical Science, Cheeloo College of Medicine, Shandong University, Jinan, Shandong P. R. China; 2 Key Laboratory of Infection and Immunity of Shandong Province, School of Basic Medical Science, Cheeloo College of Medicine, Shandong University, Jinan, Shandong P. R. China; 3 Department of stomatology, The First Affiliated Hospital of USTC, Division of Life Sciences and Medicine, University of Science and Technology of China, Hefei, Anhui P. R. China; 4 Department of Pathology, The Second Hospital, Shandong University, Jinan, Shandong P. R. China; 5 Department of Hematology, Qilu Hospital, Cheeloo College of Medicine, Shandong University, Jinan, P. R. China; 6 Institute of Pathogen Biology, School of Basic Medical Sciences, Cheeloo College of Medicine, Shandong University, Jinan, Shandong P. R. China

**Keywords:** Cancer epigenetics, DNA methylation, Gastric cancer

## Abstract

KDM6B (Lysine-specific demethylase 6B) is a histone lysine demethyltransferase that plays a key role in many types of cancers. However, its potential role in gastric cancer (GC) remains unclear. Here, we focused on the clinical significance and potential role of KDM6B in GC. We found that the KDM6B expression is upregulated in GC tissues and that its high expression in patients is related to poor prognosis. KDM6B ectopic expression promotes GC cells’ proliferation and metastasis, while its inhibition has opposite effects in vitro and in vivo. Mechanistically, KDM6B promotes GC cells proliferation and metastasis through its enzymatic activity through the induction of H3K27me3 demethylation near the CXCR4 (C-X-C chemokine receptor type 4) promoter region, resulting in the upregulation of CXCR4 expression. Furthermore, *H. pylori* was found to induce KDM6B expression. In conclusion, our results suggest that KDM6B is aberrantly expressed in GC and plays a key role in gastric carcinogenesis and metastasis through CXCR4 upregulation. Our work also suggests that KDM6B may be a potential oncogenic factor and a therapeutic target for GC.

## Introduction

The latest statistics show that nearly half of new and fatal cases of gastric cancer worldwide occur in China every year. Gastric cancer remains in the third place in terms of incidence spectrum, and seriously threatens the health of Chinese people [1]. Although the incidence and mortality rates of gastric cancer have decreased due to improvements in diet and medical conditions, it is expected that more cases will be seen in the clinic due to the aging population [2]. Since the early symptoms of gastric cancer are not obvious, more than 70% of patients are already at an advanced stage when detected, at which point, the overall prognosis of patients is unsatisfactory and the five-year survival rate is low, even if the primary focus is removed [3]. Therefore, it is of great clinical significance to explore the pathogenesis of gastric cancer and find effective therapeutic targets through basic researches.

Epigenetic modifications do not alter the DNA sequence but can affect the expression of genetic information by altering the chromatin state, which can also be inherited. Studies have shown that epigenetic regulatory disorders can lead to the development of several diseases, including tumors [4]. Histone modifications are epigenetic modifications that play important roles in the transcriptional regulation of genes, and in promoting tumorigenesis by repressing the expression of tumor suppressor genes or by accelerating the expression of oncogenes [5]. KDM6A (UTX) and KDM6B are the only two histone demethylases known to target H3K27 and that can antagonize the methylation and function of EZH2 (KMT6) on H3K27. Notably, KDM6A is structurally expressed in tissues, whereas KDM6B is expressed at relatively low levels in tissues. KDM6B (lysine-specific demethylase 6B) is a member of JmjC histone demethylases, which specifically demethylates lysine demethylation and trimethylation at position 27 of H3 protein and regulates the expression of related genes [6]. In addition, KDM6B can participate in interactions between chromatin-modifying proteins in an enzyme activity-independent manner, which activates the expression of target genes [7]. Under normal conditions, KDM6B is expressed at low levels in tissues, but many extracellular stimuli can induce its overexpression under certain abnormal conditions [8–11]. It is also induced by a variety of inflammatory factors, cellular stresses (metabolic, toxic, and tumorigenic) and small molecule compounds [8, 9, 12–14]. KDM6B plays a critical role in development, physiology, and in a variety of diseases, including tumors [7, 11, 15]. Previous studies have shown that KDM6B has a dual role in different tumor settings, by acting as a tumor suppressor and an oncogene. It was found that KDM6B can inhibit breast cancer metastasis by regulating the Wnt/β-linked protein signaling pathway [16]; while in ovarian cancer, KDM6B promotes cancer cell migration and invasion by inducing the expression of transforming growth factor β1 [17]. It has been shown that KDM6B can regulate LDHA expression through a histone demethylase activity, and thus promoting lung metastasis in osteosarcoma [18]. In prostate cancer, KDM6B exerts an oncogenic effect through the regulation of H3K27me3 levels in the CCND1 promoter region [19]. KDM6B is upregulated in hepatocellular carcinoma and clear cell renal cell carcinoma, leading to SLUG overexpression and the promotion of tumor cell proliferation and metastasis [20, 21]. On the other hand, KDM6B exerts an antitumor effect by promoting neuroblastoma differentiation [22]. The NOTCH target gene CSL promotes squamous cell carcinoma progression by suppressing the expression of KDM6B [23]. Although the role and mechanisms of KDM6B have been studied in various cancers, its function and the associated mechanism in gastric carcinogenesis and progression, are still not fully elucidated.

CXCR4 belongs to the CXCR class and is a receptor for the chemokine SDF-1 (stromal cell derived factor-1, SDF-1), a highly conserved G protein-coupled 7 transmembrane receptor encoding 352 amino acids. The interaction of CXCR4 with its specific ligand SDF-1 is important for embryonic, vascular and carcinogenesis, post-transplantation hematopoietic stem cell homing, and in mediating the transmigration of inflammatory cells, the co-stimulation of T lymphocyte proliferation, and in inflammatory response [24–27]. The aberrant expression of CXCR4 is also present in most human malignancies. It has been found that the CXCR4/SDF-1 axis promotes the development of many cancers, including colorectal, hepatocellular, and gastric cancers [28–31].

In this study, we found that KDM6B was highly expressed in human gastric cancer samples and correlated with poor prognosis in gastric cancer patients. KDM6B was found to promote the proliferation and metastasis of gastric cancer cells in vitro and in vivo via CXCR4. Our investigation of the related molecular mechanism revealed that KDM6B promotes CXCR4 expression by decreasing H3K27me3 levels, in proximity to the CXCR4 promoter region. This event was found to promote the proliferation and metastasis of gastric cancer cells. In addition, we found that *H. pylori* can induce the expression of KDM6B in vitro. The results of this study provide a research basis for finding new targets for gastric cancer.

## Results

### KDM6B is upregulated in GC and its high expression predicts a poor GC prognosis

**Fig. 1:**
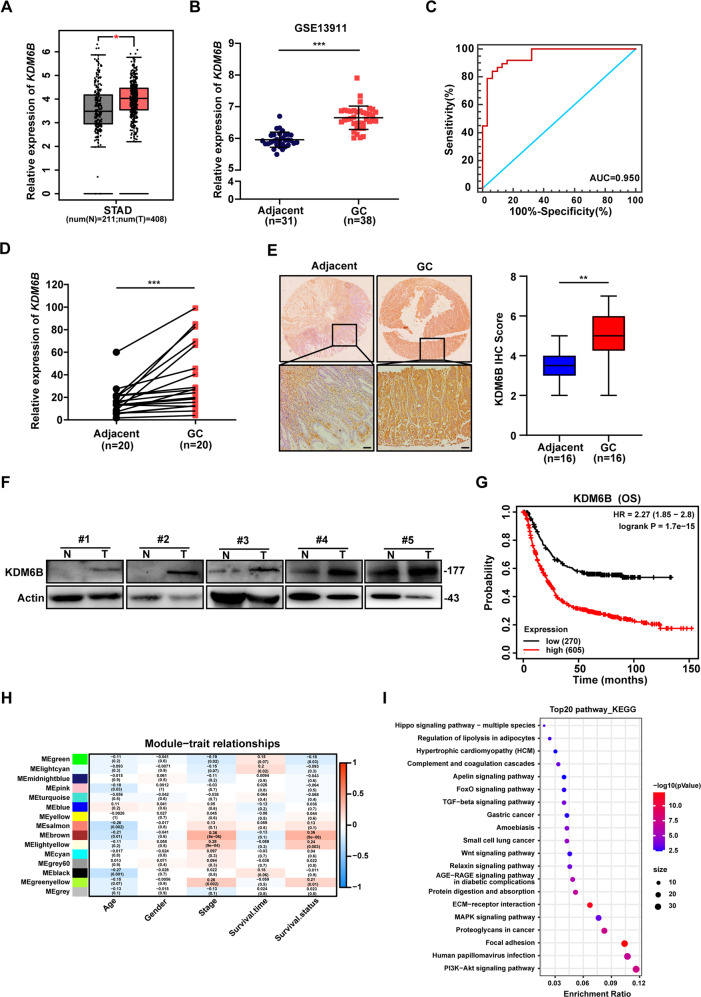
KDM6B expression is upregulated in GC tissues, which predicts a poor diagnosis and outcome in GC patients. **A** Box plots showing the difference in KDM6B expression between gastric cancer and normal tissues in the GEPIA database. **B** GSE13911 dataset analysis of the difference in KDM6B expression in gastric and adjacent tissues. **C** The efficacy of KDM6B in differentiating gastric cancer from adjacent tissues was assessed using ROC curves in the GSE13911 dataset. **D** RT-qPCR analysis of KDM6B mRNA expression in 20 clinical pairs of gastric cancer tissues and paraneoplastic tissues. **E** Immunohistochemical staining for KDM6B protein expression in human gastric cancer and its corresponding paraneoplastic tissue samples (scale insertion of 200 μm). The right panel shows the immunohistochemical correlation score. **F** Western blot detection of KDM6B protein expression in gastric cancer tissues and adjacent tissues. **G** The correlation between overall survival of GC patients and KDM6B expression levels was analyzed using Kaplan–Meier curves. **H** Heatmap of the relationship between modules and clinical features. Each cell represents the correlation analysis of gene modules with the corresponding clinical features, containing the corresponding correlation coefficients and *p*-values, with shades of color representing the magnitude of the correlation. The red indicates a positive correlation, and the blue indicates a negative correlation. **I** The brown module genes, including KDM6B, were enriched by KEGG pathway. *p < 0.05, **p < 0.01 and ***p < 0.001.

We first analyzed the expression of KDM6B in the GEPIA and dataset GSE13911 and found that KDM6B was highly expressed in gastric cancer tissues compared with that in normal or adjacent tissues (Fig. 1A, B). The analysis of the corresponding receiver operating characteristic (ROC) curve showed that KDM6B had a good performance in distinguishing gastric cancer tissues from adjacent tissues, as reflected by the area under the curve (AUC) which was 0.950 (Fig. 1C). We performed RT-qPCR on 20 pairs of gastric cancer tissues and adjacent tissues that were collected from the clinic, and the results showed that KDM6B expression was upregulated in gastric cancer (Fig. 1D). The results of immunohistochemical staining further confirmed that KDM6B expression is elevated in gastric cancer (Fig. 1E). Western blot analysis also showed that the protein expression level of KDM6B in gastric cancer tissues is significantly higher than that in the corresponding adjacent tissues (Fig. 1F). All of the above analyses and assays indicated that KDM6B was highly expressed in gastric cancer tissues. Kaplan-Meier analysis showed that a high level of KDB6B expression is significantly associated with low overall survival in gastric cancer patients (Fig. 1G). These results strongly suggest that KDM6B has an oncogenic role in GC.

To explore the biological functions of KDM6B in gastric cancer, we performed WGCNA analysis using the gastric cancer dataset GSE15460. Firstly, the transcriptome data of 248 clinical samples were corrected, and then the hierarchical clustering tree and the clinical characteristic heat map (including Age, Gender, Stage, Survival time, and Survival status), were constructed. The cut height was set to 120 to eliminate three obviously outlier samples GSE387958, GSE387843, and GSE387854 (Supplementary Fig. 1A). To increase the consistency of the constructed gene network with the scale-free network characteristics, we selected β = 5 as the soft threshold for this study (Supplementary Fig. 1B), at which the fitting coefficient R2 was 0.92 and the slope was −1.38. The results of this analysis met the requirements of the scaled network distribution (Supplemental Fig. 1C). As a result, 14 modules of co-expressed genes were identified after excluding gray modules using merged dynamic tree cuts (Supplementary Fig. 1D). We analyzed the correlation between the gene modules and the clinical features, and found that three modules, the brown module, the light-yellow module, and the green module were positively correlated with tumor stage and negatively correlated with patient survival (Fig. 1H). Using the KEGG pathway, we enriched the genes in the brown module of KDM6B by, and the results showed that many tumors and signaling pathways that are related to carcinogenesis progression, such as gastric cancer, Wnt signaling pathway, MAPK signaling pathway and PI3K Akt signaling pathway, were enriched (Fig. 1I).

In conclusion, these results strongly suggest that KDM6B has an oncogenic role in gastric cancer.

### KDM6B promotes GC cells proliferation in vitro and tumor formation in vivo

**Fig. 2:**
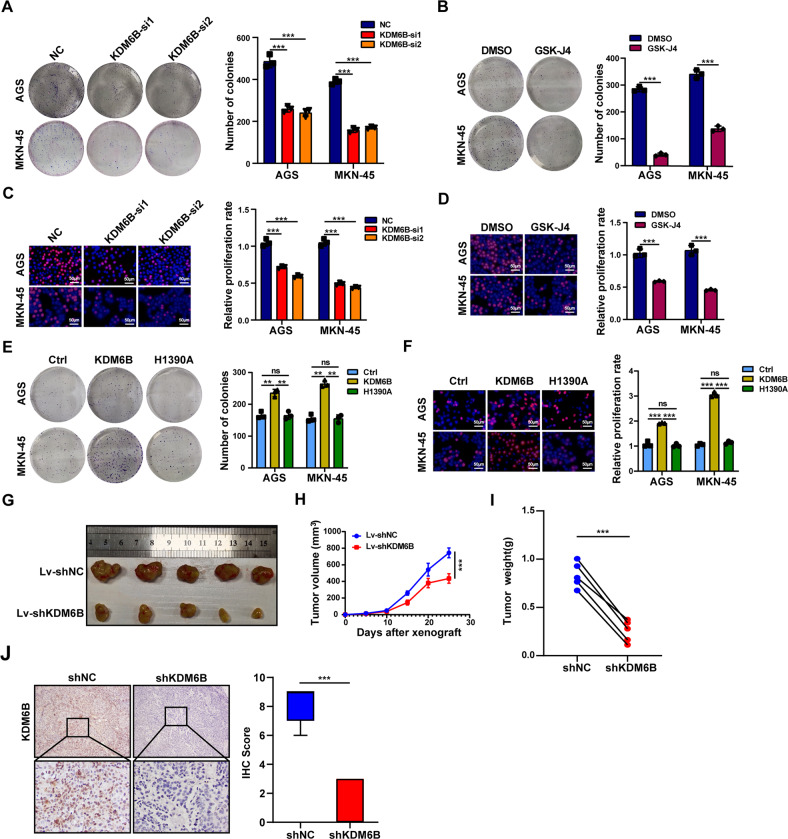
KDM6B promotes the proliferation of gastric cancer cells. **A** After transfection of siKDM6B and NC (negative control) in gastric cancer cells, the cells clone-forming ability was examined, and the bar graph on the right is the statistical result. **B** Gastric cancer cells were transfected with the KDM6B inhibitor, GSK-J4, and DMSO to detect the ability of the cells’ clone formation. The graph bar on the right is the statistical result. **C** After transfection of siKDM6B and the negative control NC in gastric cancer cells, cell proliferation was detected by the EdU assay, and the bar graph on the right is the statistical result. **D** Cell proliferation was detected by the EdU after the addition of the KDM6B inhibitor, GSK-J4, and DMSO in gastric cancer cells. The graph bar on the right is the statistical result. **E** The ability of clone formation was detected after transfection of the gastric cancer cells with KDM6B plasmids (WT or H1390A). The graph bar on the right is the statistical result. **F** Cell proliferation was detected by the EdU assay after transfection of in gastric cancer cells with KDM6B plasmids (WT or H1390A). The bar graph on the right is the statistical result. **G** Images of subcutaneous tumors in two groups. **H** In vivo tumor growth curves were plotted. **I** Comparative statistics of tumor weights of the two groups of mice. **J** IHC staining to detect the expression of KDM6B in xenograft tumors. **p < 0.01, ***p < 0.001, ns: not significant.

Next, we explored the role of KDM6B in gastric progression. The results of the colony formation assay showed that the clonogenic ability of GC cells significantly decreases after KDM6B knockdown (Fig. 2A and Supplementary Fig. 2A, B). KDM6B is a histone demethylase, and many studies have shown that the biological function of KDM6B is often closely related to its enzymatic activity. To investigate whether the biological function of KDM6B in gastric cancer depends on its enzymatic activity, we added 1 μM of GSK-J4, an inhibitor of the enzymatic activity of KDM6B, to gastric cancer cells, and the results showed that 1 μM GSK-J4 significantly inhibits the clonogenic ability of gastric cancer cells (Fig. 2B). The results of EdU assays showed that both KDM6B knockdown and the addition of the inhibitor GSK-J4 significantly reduce the proliferation of gastric cancer cells (Fig. 2C, D). In addition, the results of clonogenic assay also showed that the clonogenic ability of gastric cancer cells is significantly enhanced after the ectopic expression of KDM6B compared with the control, but the clonogenic ability of GC cells transfected with the KDM6B enzymatic activity-deficient mutant plasmid (H1390A) did not change (Fig. 2E, and Supplementary Fig. 2C, D). The results of EdU assay also showed that the ectopic expression of KDM6B significantly enhances the proliferation ability of GC cells, while the KDM6B enzyme activity-deficient mutant (H1390A) had no effect (Fig. 2F).

To further investigate the effect of KDM6B on GC cells growth in vivo, we subcutaneously injected cells that stably carry the construct, knockdown Lv-shKDM6B-MKN-45 or its negative control Lv-shNC-MKN-45, into the anterior left and right sides of the axilla of Balb/c nude mice, respectively. The in vivo imaging results of the mice showed that the tumor volume on the side injected with Lv-shKDM6B-MKN-45 cells was significantly smaller than that on the negative control side (Fig. 2G and Supplementary Fig. 2E, F). The growth curve showed that the tumor growth rate on the side injected with Lv-shKDM6B-MKN-45 cells was smaller than that on the negative control side (Fig. 2H). The tumor weights on the Lv-shKDM6B side were significantly lower than those on the negative control side (Fig. 2I). The IHC staining showed that the expression of KDM6B in shKDM6B group was much lower than the negative control group (Fig. 2J). These results suggest that KDM6B can promote GC cells growth in vitro and in vivo.

### KDM6B promotes gastric cancer cells’ migration in vitro and in vivo

**Fig. 3:**
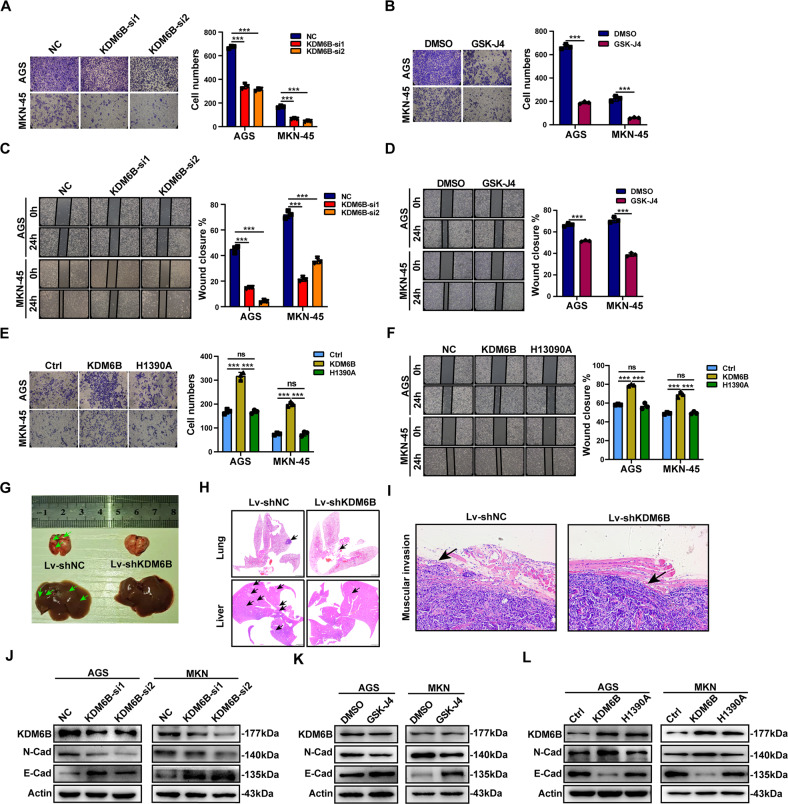
KDM6B promotes metastasis of gastric cancer cells. **A** Transwell assay to detect the cell migration after transfection of AGS and MKN-45 cells with KDM6B siRNA. The graph bar on the right shows the statistical results. **B** Transwell assay to detect the metastatic ability of AGS and MKN-45 cells after the addition of GSK-J4. The graph bar on the right shows the statistical results. **C** Scratch assay to detect the transfer ability of AGS and MKN-45 cells after transfection with KDM6B siRNA. The graph bar on the right shows the statistical results. **D** Scratch assay to detect the migration of AGS and MKN-45 cells after addition of GSK-J4. The graph bar on the right shows the statistical results. **E** The metastatic ability of cells was detected by the transwell assay after transfection of the gastric cancer cells with KDM6B plasmids (WT or H1390A). The graph bar on the right shows the statistical results. **F** After transfection of the gastric cancer cells with KDM6B plasmids (WT or H1390A), the migration of the cells was detected by scratch assay. The graph bar on the right is the statistical result. **G** Nude mice were dissected, and lung and liver metastases were visually observed (the green arrows indicate metastatic nodules). **H** Number and size of lung metastases in nude mice observed in panoramic scans of H&E-stained sections of lung and liver (black arrows indicate metastatic nodules). **I** The H&E staining was performed to observe the integrity of the tumor envelope and local infiltration in the two groups of mice. **J** The protein expressions of three EMT markers were detected after knockdown of KDM6B in AGS and MKN-45 gastric cancer cells. **K** The protein expressions of three EMT markers were detected in AGS and MKN-45 gastric cancer cell lines after addition of GSK-J4. **L** The protein expressions of three EMT markers were detected after transfection of the gastric cancer cells, AGS and MKN-45, with KDM6B plasmids (WT or H1390A). ***p < 0.001, ns: not significant.

Since the occurrence of metastasis is a major feature of gastric cancer progression, we explored the biological function of KDM6B in gastric cancer cells’ migration. The results of the transwell assay showed that the migration of GC cells is not inhibited by KDM6B knockdown or by the addition of GSK-J4 when compared with the control group (Fig. 3A, B). The results of the wound healing assay showed similar results as reflected by the absence of inhibition of wound healing of GC with KDM6B knockdown or when GSK-J4 was added (Fig. 3C, D). In contrast, of the ectopic expression of KDM6B promoted migration and wound healing in GC cells, but the ectopic expression of the KDM6B had no effects (Fig. 3E, F).

To investigate the effect of KDM6B on the migration ability of GC cells in vivo, we injected the cells that stably carry the construct, knockdown Lv-shKDM6B-MKN-45 or its negative control Lv-shNC-MKN-45 into nude mice via the tail vein to establish an in vivo tumor metastatic model. The results showed that KDM6B knockdown reduces the number of hepatopulmonary metastatic nodules in gastric cancer cells compared with that in the control group (Fig. 3G). The results of H&E-stained liver and lung sections showed that KDM6B knockdown reduces the volume of lung metastatic nodules and decreases the number of liver metastatic nodules compared with that in the control group (Fig. 3H). The H&E staining of xenograft tumors revealed that the tumor tissue envelope was intact on the Lv-shKDM6B side and without obvious local infiltration, while the tumor tissue envelope was disrupted and with local infiltration on the control side (Fig. 3I). All of these results revealed that KDM6B promoted gastric cancer cells’ migration in vivo.

Since epithelial mesenchymal transition (EMT) underlies the ability of tumors to migrate, we examined the effect of KDM6B on GC cells’ EMT markers. Western blot results showed that KDM6B knockdown or the addition of GSK-J4 decreases the protein expression level of the mesenchymal marker, N-cadherin and increases the protein expression level of the epithelial-associated markers, E-cadherin when compared with controls (Fig. 3J, K). In contrast, KDM6B ectopic expression resulted in an increase in the protein expression level of N-cadherin and a decrease in the protein expression level of E-cadherin when compared with controls. However, the levels of both proteins were not significantly altered in cells overexpressing the KDM6B mutant (Fig. 3L).

Taken together, we confirmed that KDM6B promotes GC cells’ migration in vitro and in vivo and that this effect is dependent on its enzymatic activity.

### CXCR4 is a target gene downstream of KDM6B

**Fig. 4:**
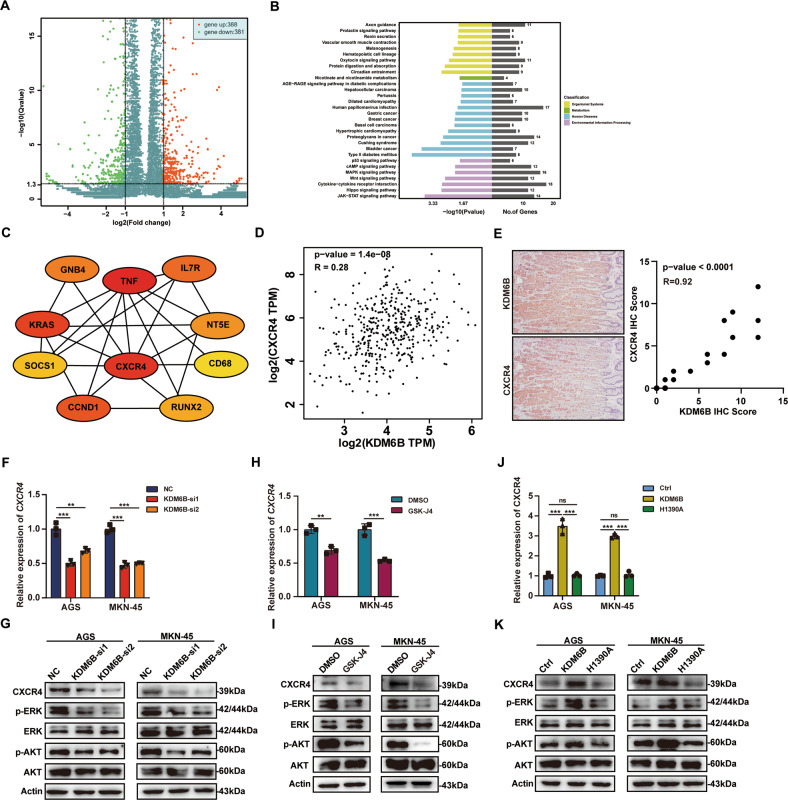
CXCR4 is a target gene downstream of KDM6B. **A** Volcano plot showing the differential gene expression obtained by RNA-Seq after KDM6B knockdown (|log2 (Fold Change | )>1, Q value < 0.05). **B** Histogram of differential gene pathway enrichment analysis obtained by RNA-Seq. **C** Relationship plot of genes in the top 10 of MCC scores in RNA-Seq differential gene protein interaction network. **D** Scatter plot showing the relationship map of KDM6B and CXCR4 mRNA expression obtained from GEPIA data. **E** Immunohistochemical staining for KDM6B and CXCR4. Scatter plot showing the relationship map of KDM6B and CXCR4 IHC Score. **F** RT-qPCR was performed to detect CXCR4 mRNA levels after KDM6B siRNA transfection. **G** Western blot was performed to detect the protein expression levels of CXCR4, AKT, p-AKT, ERK, and p-ERK after transfection of the cells with KDM6B siRNA. **H** RT-qPCR was performed to detect CXCR4 mRNA levels after addition of the KDM6B inhibitor, GSK-J4. **I** Western blot was performed to detect the protein expression levels of CXCR4, AKT, p-AKT, ERK, and p-ERK after addition of the KDM6B inhibitor, GSK-J4. **J** RT-qPCR was performed to detect CXCR4 mRNA levels after addition transfection of the cells with KDM6B plasmids (WT or H1390A). **K** Western blot was performed to detect the protein expression levels of CXCR4, AKT, p-AKT, ERK, and p-ERK after addition transfection of the cells with KDM6B Plasmids (WT or H1390A). **p < 0.01, ***p < 0.001, ns: not significant.

To further investigate the specific mechanisms underlying the role of KDM6B in gastric cancer, we performed RNA-Seq on MKN-45 cells with KDM6B knockdown and the negative control (NC) cells. We found that KDM6B knockdown results in altered expression of 769 genes (Fig. 4A). Next, we performed pathway enrichment analysis of genes with altered expressions and found that multiple tumors and tumor-related signaling pathways were enriched, such as in Gastric cancer, Breast cancer, Bladder cancer, and signaling pathways, including the p53 signaling pathway, the MAPK signaling pathway, the Wnt signaling pathway, the Hippo signaling pathway, and the JAK-STAT signaling pathway. These results indicate that the dysregulation of KDM6B expression affects numerous tumors and signaling pathway genes associated with tumor progression (Fig. 4B). To further search for core genes that are regulated by KDM6B, we performed an analysis of protein-protein interactions on the above 769 genes using the platform “https://www.string-db.org/” and entered the results into Cytoscape for maximum cluster centrality (MCC) scoring, using the cytoHubba plugin. From this approach, we obtained a network graph of the top 10 scoring genes, with CXCR4 located at the center of this network graph (Fig. 4C). Previous studies have shown that CXCR4 plays an important role in the development of many tumors, including gastric cancer [28, 32], and thus, we next focused on CXCR4. We analyzed the mRNA expression levels of KDM6B and CXCR4 in GEPIA’s gastric cancer database and found that they were positively correlated (Fig. 4D). We also analyzed the IHC Score of KDM6B and CXCR4 in gastric cancer tissues and found that they were positively correlated, too (Fig. 4E). RT-qPCR and western blot assays showed that KDM6B knockdown or the addition of GSK-J4, downregulates CXCR4 mRNA and protein expression (Fig. 4F–I). In turn, KDM6B ectopic expression promoted CXCR4 mRNA and protein expression in GC cells, whereas its mutant (H1390A) did not significantly show effects (Fig. 4J, K). Previous studies have shown that CXCR4 promotes tumor cell proliferation and migration, mostly through its downstream ERK1/2 and AKT pathways [33, 34], and therefore, we examined whether the dysregulation of KDM6B expression affects the ERK and AKT pathways in GC cells. The results showed that KDM6B knockdown or the addition of the inhibitor GSK-J4 downregulates the levels of p-ERK and p-AKT (Fig. 4G, I), and that the ectopic expression KDM6B upregulates p-AKT and p-ERK levels. However, the ectopic expression of the KDM6B mutant had no effects (Fig. 4K).

The above results suggest that CXCR4 may be a potential downstream target gene of KDM6B and that the regulation of CXCR4 expression by KDM6B depends on its enzymatic activity.

### KDM6B regulates CXCR4 expression through H3K27me3

**Fig. 5:**
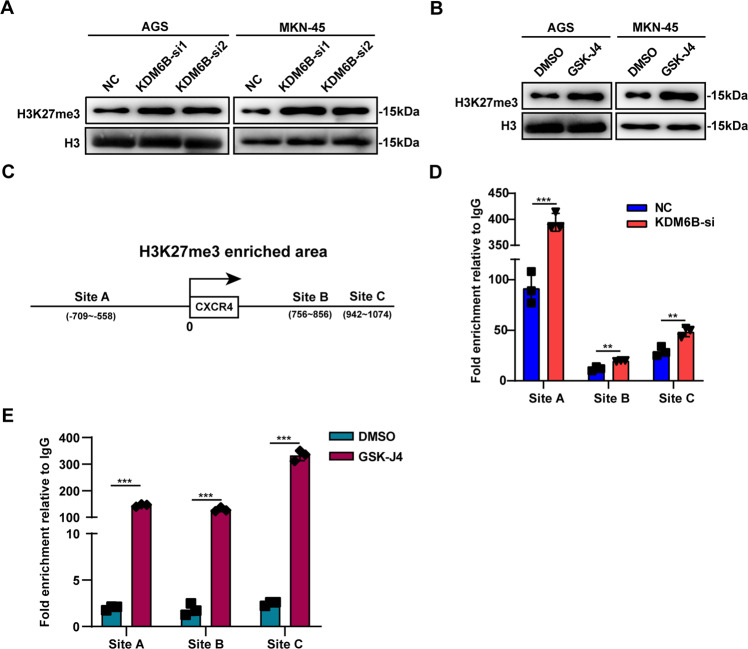
KDM6B affects CXCR4 expression through H3K27me3. **A** Western blot detection of cellular H3K27me3 protein levels in AGS and MKN-45 cells after transfection with KDM6B siRNA. **B** Western blot detection of H3K27me3 protein levels in AGS and MKN-45 cells after treatment with GSK-J4. **C** Pattern diagram showing three primer amplification regions designed upstream and downstream of the CXCR4 promoter region in ChIP-qPCR experiments. **D** The enrichment level of H3K27me3 in the upstream and downstream of the CXCR4 promoter region was detected by ChIP-qPCR assay after transfection of MKN-45 cells with KDM6B siRNA. **E** The enrichment level of H3K27me3 upstream and downstream of the CXCR4 promoter region was detected by ChIP-qPCR assay after addition of the KDM6B inhibitor GSK-J4 in MKN-45 cells. **p < 0.01 and ***p < 0.001.

Previous studies have shown that KDM6B is an important histone demethylase in cells, which mainly regulates downstream genes through demethylation of H3K27me3. Therefore, to further explore the role of KDM6B enzymatic activity in GC cells, we extracted histones from GC cells for analysis. Western blot results showed that KDM6B knockdown or addition of its enzyme activity inhibitor, GSK-J4, upregulates the protein level of H3K27me3 in GC cells compared with that in the control group (Fig. 5A, B). Based on the experimental conclusion mentioned in this article, we hypothesized that KDM6B may regulate the expression of CXCR4 by controlling the level of H3K27me3 near the CXCR4 promoter region through its enzymatic activity. To confirm this, we used the WashU website (https://epigenomegateway.wustl.edu/) to predict the enrichment region of H3K27me3 near the CXCR4 transcription start site. We found that there were H3K27me3 enrichment regions upstream and downstream of the CXCR4 transcription start site. Therefore, we designed three pairs of primers in three enrichment regions (Fig. 5C) and performed ChIP-qPCR. The results showed that KDM6B knockdown or the addition of its enzyme activity inhibitor GSK-J4 significantly increased the enrichment of H3K27me3 near the CXCR4 promoter region in GC cell MKN-45 and when compared with the control group (Fig. 5D, E).

These results suggest that KDM6B can change the enrichment of H3K27me3 near the CXCR4 promoter region through its enzyme activity, leading to the regulation of CXCR4 expression in GC cells.

### KDM6B promotes gastric cancer cell proliferation and migration dependent on CXCR4

**Fig. 6:**
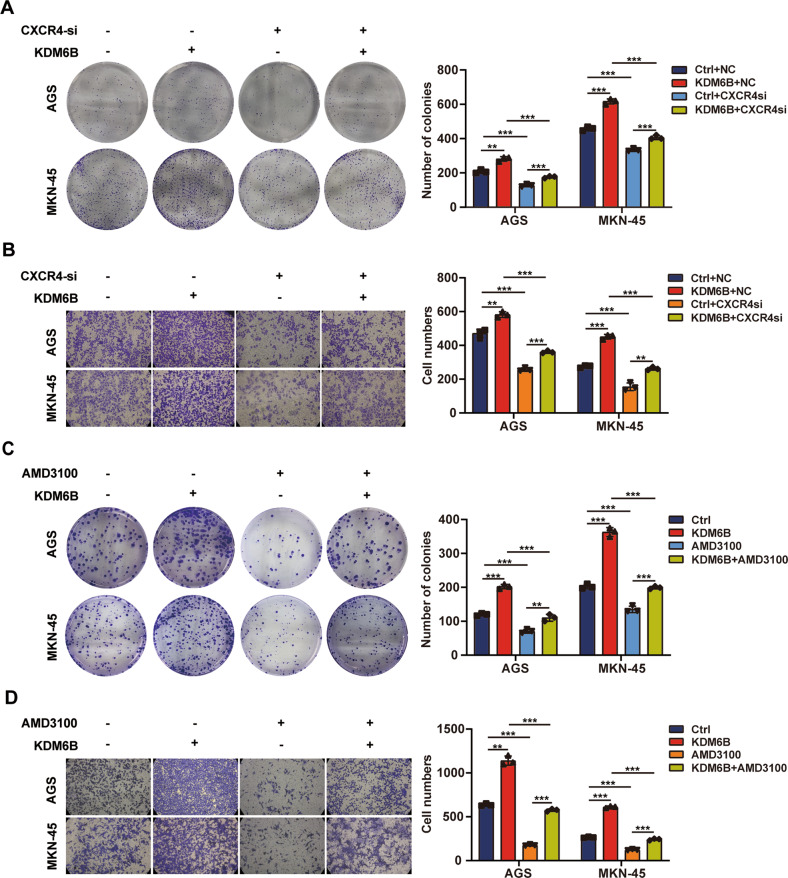
KDM6B affects gastric cancer cell proliferation and migration via CXCR4. **A** Clone formation assay was used to detect the proliferation of the cells after transfection with KDM6B plasmid and CXCR4 siRNA in AGS and MKN-45. **B** Transwell assay to detect cell migration after transfection of AGS and MKN-45 cells with KDM6B plasmid and CXCR4 siRNA. **C** Clone formation assay to detect cell proliferation after transfection of AGS and MKN-45 cells with KDM6B plasmid and the addition of the CXCL12/CXCR4 signaling pathway inhibitor, AMD3100. **D** Transwell assay to detect cell migration after transfection of in AGS and MKN-45 cells with KDM6B plasmid and the addition of the CXCL12/CXCR4 signaling pathway inhibitor, AMD3100. **p < 0.01 and ***p < 0.001.

The above results revealed that KDM6B could directly regulate the expression of CXCR4. We first constructed CXCR4 knockdown cells and found that CXCR4 mRNA and protein levels were significantly reduced after CXCR4 knockdown (Supplementary Fig. 3A, B). We next performed rescue assay on the proliferation and migration of gastric cancer cells. The results of the clone formation and transwell assays showed that CXCR4 inhibition by siRNA, reverts the increase in cell proliferation and migration caused by KDM6B ectopic expression (Fig. 6A, B). CXCR4 is the receptor of CXCL12, and the CXCL12/CXCR4 signaling pathway plays an important role in the occurrence and metastasis of a variety of tumors. Therefore, we investigated whether KDM6B plays a cancer promoting role in gastric cancer cells that depends on the CXCL12/CXCR4 signaling pathway. The results of clone formation and transwell assays showed that the addition of the CXCL12/CXCR4 signaling pathway by the inhibitor, AMD3100, reverses the increase in the proliferation and migration of gastric cancer cells that was caused by KDM6B ectopic expression (Fig. 6C, D).

The above results suggest that the role of KDM6B in promoting cell proliferation and migration in GC cells is largely dependent on the CXCL12/CXCR4 signal pathway.

### *Helicobacter pylori* infection promotes the expression of KDM6B

**Fig. 7:**
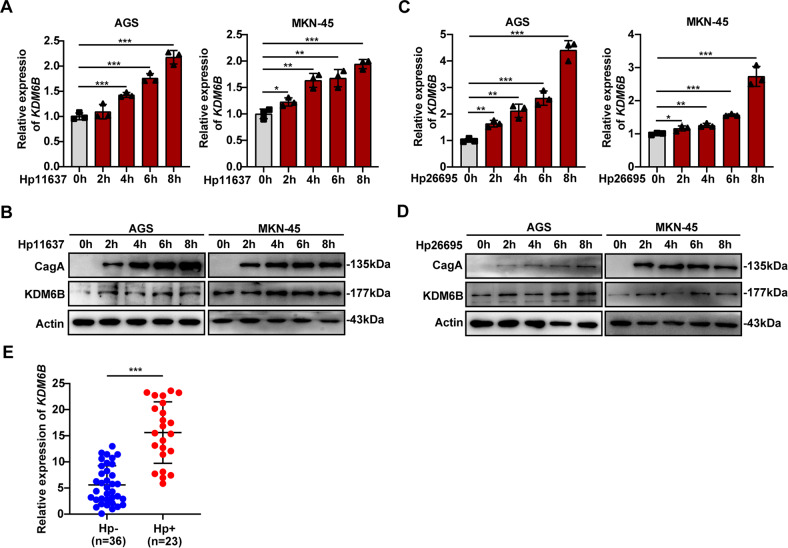
*H. pylori* infection upregulates the expression of KDM6B. **A** RT-qPCR detection of KDM6B mRNA expression after infection of the gastric cancer cell lines, AGS and MKN-45 with H. pylori (*Hp11637*) for the indicated times. **B** Western blot detection of KDM6B and CagA protein expressions in the gastric cancer cell lines, AGS and MKN-45, after infection with *H. pylori* (*Hp11637*) for the indicated times. **C** RT-qPCR detection of KDM6B mRNA expression in the gastric cancer cell lines, AGS and MKN-45, after infection with H. pylori (*Hp26695*) for the indicated times. **D** Western blot detection of KDM6B and CagA protein expressions in the gastric cancer cell lines, AGS and MKN-45, after infection with *H. pylori* (*Hp26695*) for the indicated times. **E** RT-qPCR was performed to detect KDM6B mRNA levels in H. pylori-negative or H. pylori-positive human GC samples. *p < 0.05, **p < 0.01 and ***p < 0.001.

Since *H. pylor*i infection is a major risk factor for the development of gastric cancer, we explored whether the expression of KDM6B was associated with *H. pylori* infection. We used *H. pylori* strains (*Hp11637* and *Hp26695*) to infect the gastric cancer cells, AGS and MKN-45, at different time points (2 h, 4 h, 6 h, and 8 h) based on a 100:1 ratio of bacteria to cells, and then collected the cells to detect the expression of KDM6B. The results of RT-qPCR and western blot assay showed that KDM6B mRNA and protein expressions in AGS and MKN-45 cells increase with the increase in time of bacterial infection (Fig. 7A–D). We also detected KDM6B mRNA levels in H. pylori-negative or H. pylori-positive human GC samples. KDM6B mRNA levels were higher in H. pylori-positive human GC samples (Fig. 7E). These results suggest that *H. pylori* can promote the expression of KDM6B in GC cells and GC patients.

## Discussion

According to the statistics, more than half of the world’s population is infected with *H. pylori*. *H. pylori*, as the Class I carcinogen of gastric cancer, plays a vital role in inducing the disorder of gene expression and regulation, which is the internal driving force of disease occurrence and development. Previous studies showed that *H. pylori* and its virulence factors act on gastric epithelial cells, activating tumorigenic related signal pathways, such as MAPK, NF-κB, JNK, etc., promote the malignant transformation of gastric epithelial cells. It was previously reported that porcine circovirus type 2 can regulate JMJD3 expression via activating NF-κB and JNK signaling pathways and its structure protein Cap can directly bind to the KDM6B promoter in nucleus, which eventually alter the histone suppressive mark H3K27me3 [35]. Therefore, it is reasonable to infer that *H. pylori* can regulate the expression of KDM6B via activating tumorigenic related transcription factors such as c-jun by virulence factors CagA and VacA. More experiments are needed to further explore this inference.

In this study, we found that KDM6B is highly expressed in gastric cancer tissues through the analysis of gastric cancer datasets and clinical samples. We found that the prognosis of patients with high KDM6B expression was poor. The results showed that KDM6B promotes the proliferation and metastasis of gastric cancer cells in vitro and in vivo, and that this effect depends on its enzymatic activity. To identify the downstream target genes of KDM6B in gastric cancer, we compared differentially expressed genes between MKN-45 and MKN-45 cells following KDM6B knockdown using transcriptome sequencing and identified a series of upregulated and downregulated genes. Finally, we selected CXCR4 as a downstream target gene of KDM6B. Previous studies have shown that gastric cancer patients with high expression of CXCR4 have a poor prognosis and therefore, CXCR4 can be used as a prognostic marker of gastric cancer [36, 37]. CXCR4 is a stem cell marker of GC cells and promote their proliferation and metastasis [36–41]. Further studies showed that the knockdown or addition of the KDM6B enzyme activity inhibitor, GSK-J4, can downregulate the expression of CXCR4 in gastric cancer cells. KDM6B ectopic expression upregulated the expression of CXCR4 in GC cells, while of the ectopic expression of the KDM6B mutant (H1390A) did not affect CXCR4 expression. ChIP-qPCR results indicated that KDM6B regulates the expression of CXCR4 through its demethylase activity. In addition, we also found that *H. pylori* can promote the expression of KDM6B, which is consistent with the observations regarding the induction of KDM6B expression by the above endogenous or exogenous substances.

**Fig. 8:**
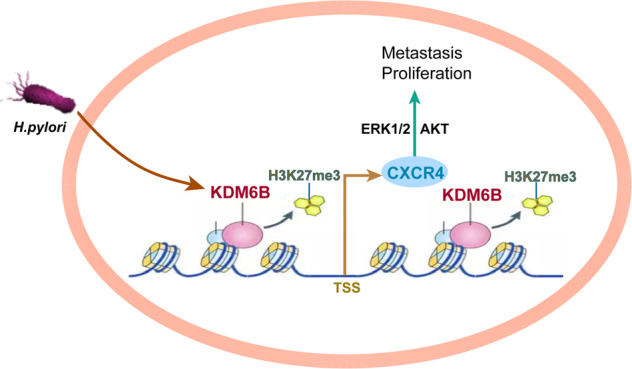
Model diagram of the mechanism of KDM6M in promoting gastric carcinogenesis and progression. Helicobacter pylori promotes the expression of KDM6B, which upregulates CXCR4 expression through its enzymatic activity leading to H3K27me3 demethylation upstream and downstream of the CXCR4 promoter region, resulting in the promotion of the proliferation and metastasis of gastric cancer cells.

In summary, we found that *H. pylori* can induce the expression of KDM6B and promote the demethylation of H3K27me3 near the CXCR4 promoter region through its enzymatic activity, resulting in the upregulation of CXCR4 expression. CXCR4 ectopic expression activates the ERK1/2 and AKT signaling pathways and causes the proliferation and metastasis of GC cells (Fig. 8). In conclusion, we experimentally confirmed that KDM6B has an oncogenic function in gastric cancer and that it can be used as a potential target for gastric cancer therapy.

## Materials and methods

### Dataset download and processing

Download GSE13911 (*n* = 38) [42], GSE15460 (*n* = 248) [43] gastric cancer transcriptional data on the GEO website (https://www.ncbi.nlm.nih.gov/), where the GSE15460 dataset contains clinical data. The “affy” and “impute” packages were used in R software (version 3.6.3) for background processing, normalization and log2 transformation; the “limma “package for differential gene analysis between tumor and normal groups. The survival curves of KDM6B used for modeling were from the Kaplan-Meier plotter (http://kmplot.com/analysis/index.php?p=background) [44].

### Cell culture

AGS cells were obtained from the Cell Bank of the Chinese Academy of Sciences (Shanghai, PR China). MKN-45 cells were obtained from the National Cell Line Resource Infrastructure (Beijing, PR China). AGS cells were cultured in F12 medium (HyClone, Logan, UT, USA) supplemented with 12% FBS (Gibco, Carlsbad, CA, USA). MKN-45 cells were cultured in RPMI-1640 (Gibco) containing 10% FBS. All cells were cultured in a humidified cell incubator (Thermo Fisher Scientific, Waltham, MA, USA) at 37°C, 95% air and 5% CO_2_, verified by short tandem repeat analysis and tested for absence of mycoplasma.

### Cell transfection and addition of inhibitors

Roche transfection reagent (2 μl per sample; Roche, Basel, Switzerland) was used to transfect KDM6B or KDM6B (H1390A) (Addgene, Watertown, MA, USA), as recommended. Cells were collected 48 h post-transfection for further analysis. Lipofectamine 2000 (5 μl per sample; Invitrogen, Carlsbad, CA, USA) was used to transfect siRNA (5 μl per sample) into GC cells (4 × 10^5^ cells per sample) as recommended. Cells were collected 72 h post-transfection. GC cells were inoculated into 24-well plates and 24 h later, the virus (containing shKDM6B or shNC; Genechem, Shanghai, PR China) was transfected into GC cells with a complex of infection (MOI) of 100. Polybrene (Genechem) was used to increase efficiency of transfection. The medium was changed 24 h after transfection and puromycin (2 μg/ml; Gibco) was added. Three days later, transfection efficiency was calculated using RT-PCR and protein blotting. The sequences of siRNAs and shRNAs used in the experiments are listed in Supplementary Material Table S1. The final concentration of KDM6B inhibitor GSK-J4 (HY-15648B, MCE, Monmouth Junction, NJ, USA) used in this study is 1 μM, and CXCR4 inhibitor AMD3100 (HY-10046, MCE) is 100 nM.

### Clinical tissue samples

10 pairs of gastric cancer tissues and paracancer tissues RNA specimens and 5 pairs of gastric cancer and corresponding paracancer tissue protein specimens were obtained from Qilu Hospital of Shandong University. 16 pairs of gastric cancer and paracancer tissue specimens for immunohistochemical staining were obtained from Jinan Central Hospital of Shandong University.

### Construction of weighted gene co-expression network analysis (WGCNA)

The GSE15460 dataset included 248 GC patients with detailed clinical information suitable for the construction of WGCNA. We can link the module characteristic gene with the clinical information of GC patients. We first constructed the data matrix of gene expression in GSE15460, and used the “WGCNA” package in R/Bioconductor software to select the top 25% genes with the largest variance in tumor samples as the input data set of subsequent WGCNA. The sample hierarchical clustering method is used to detect and eliminate abnormal samples, and then the appropriate soft threshold is selected to realize the scale-free network. In the next stage, through the construction of adjacency and topological overlap matrix (TOM) and the calculation of corresponding dissimilarity (1-TOM), the gene tree and module identification are completed by dynamic tree cutting, and then the module characteristic genes are clustered to merge highly similar modules with the dissimilarity of <0.25, and the correlation between module characteristic genes and GC clinical phenotype is calculated.

### KEGG pathway enrichment analysis

KEGG (Kyoto Encyclopedia of Genes and Genomes) pathway-related database, based on which the cellular signaling pathways in which the target genes are involved can be known. In this study, KEGG analysis of the target gene set was performed using the “ClusterProfiler” R package.

### RNA extraction and RT-qPCR

Total RNA was extracted using Trizol reagent (Invitrogen), and cDNA synthesis was performed using RT kit gDNA Eraser (Takara, Shiga, Japan). The corresponding cDNAs were analyzed by quantitative PCR using SYBR Green (Takara). The primer sequences are listed in Supplementary Material Table S2.

### H. pylori cultures

All animal experiments were reviewed and approved by the Ethics Committee of the School of Basic Medical Sciences, Shandong University. *H. pylori* strains (11637 and 26695) were grown in brucella broth supplemented with 5% FBS at 37 °C in a microaerobic environment.

### In vivo proliferation and metastasis assays

Twenty-five male thymus null BALB/c nude mice (6 weeks old) were purchased from Charles River Labs (Beijing, PR China) for the following experiments. For the tumor xenograft mouse model, MKN-45 cells (1 × 10^6^ cells) were transfected with KDM6B shRNA or negative control and injected into the right or left subcutis of 5 nude mice. Tumor growth was checked every 2 days, and mice were euthanized 14 days after injection. For the metastatic mouse model, ten male thymus null BALB/c nude mice were divided into two groups. MKN-45 cells (1 × 10^6^ cells) were transfected with KDM6B shRNA or negative control and injected intravenously into each group via the tail vein. One month after injection, the mice were euthanized, and organs were removed and photographed.

### RNA-sequencing analysis (RNA-Seq)

MKN-45 cells from the negative control group and KDM6B knockdown group were added with TRIzol and sent to Guangzhou RiboBio Co., Ltd for RNA sequencing and differential gene analysis. The results of differential gene analysis are listed in Supplementary Material Table S3.

### Chromatin Immunoprecipitation (ChIP)

ChIP is performed using the SimpleChIP® Enzymatic Chromatin IP Kit (Cell Signaling Technology, Danvers, MA, USA). GC cells are cross-linked and lysed. The lysate is sonicated to make soluble chromatin with DNA fragments. Antibodies were used to precipitate the DNA fragments. Protein-DNA complexes were collected with protein G Sepharose beads, eluted and reverse cross-linked. After treatment with Proteinase K, DNA is extracted, precipitated from the samples, and then analyzed using qPCR. The antibodies used in this study are as follows: anti-KDM6B (ab38113, Abcam, Cambridge, UK), anti-H3K27me3 (ab6002, Abcam), and anti-IgG (2729, Cell Signaling Technology). The primer sequences are listed in Supplementary Material Table S4.

### Colony formation and EdU assays

For colony formation analysis, GC cells (500–1000 cells per well) were inoculated into six-well plates and cultured for 1-2 weeks. Cells were then fixed with methanol and stained with Giemsa Staining Solution. For Edu analysis, GC cells (10,000 cells per well) are inoculated in triplicate into 96-well plates and incubated for 24 h. Then, the following experiments were performed according to the EdU (RIBOBIO, Guangzhou, PR China) kit procedure, and then photographed with a fluorescent microscope.

### Western blotting

Cell lysates were obtained by extraction with protein lysis buffer supplemented with protease inhibitors. Lysates were then resolved using SDS-PAGE. The protein gel is then transferred to a PVDF membrane and closed with 5% skim milk powder and incubated overnight at 4 °C with the primary antibody. The PVDF membrane is then incubated with the appropriate secondary antibody. Signals were analyzed using ECL detection reagents (Millipore, St. Louis, MO, USA). The antibodies used in this study are as follows: anti-KDM6B (ab169197, Abcam), anti-β-actin (60008-1, Proteintech, Wuhan, PR China), anti-CXCR4 (ab181020, Abcam), anti-E-Cadhrein (3195, Cell Signaling Technology), anti-N-Cadhrein (13116, Cell Signaling Technology), anti-AKT (4691, Cell Signaling Technology), anti-p-AKT (4064, Cell Signaling Technology), anti-ERK (4695, Cell Signaling Technology), and anti-p-ERK (4370, Cell Signaling Technology).

### Immunohistochemistry (IHC)

Formalin-fixed paraffin-embedded (FFPE) sections are prepared from mouse or patient samples and are dewaxed and rehydrated. Samples were subjected to epitope repair and H2O2 treatment, followed by closure with 3% BSA solution. Thereafter, samples are incubated overnight at 4 °C with the specific primary antibody. The next day, sections were incubated with the corresponding secondary antibodies and then analyzed using a DAB staining kit (Vector Laboratories, Burlingame, CA, USA). Positive staining intensity was scored as follows: 0 (no staining), 1 (light brown), 2 (medium brown) and 3 (dark brown). The percentage of positively stained cells was scored using a scale of 0 to 3: 0 (0%), 1 (<25%), 2 (25–75%) and 3 (>75%). the IHC score was calculated as a multiple of the above two scores. The antibodies used in this study are as follows: anti-KDM6B (Abcam, ab38113), and anti-CXCR4 (Abcam, ab181020).

### Wound healing and transwell assay

For the wound healing assay, 5 × 10^5^ cells were initially inoculated into 12-well plates and when confluent, the cell surface was scraped using a pipette tip. 24 h later, accurate wound measurements were made using the formula wound closure = (wound width at 0 h - wound width at 24 h)/wound width at 0 h ×100% to calculate wound closure. Cells were observed using an inverted phase contrast microscope and photographed.

For the migration assay, a total of 5 × 10^4^ cells were inoculated into the upper chamber of the Transwell. After 48 h, cells migrating to the lower well were fixed with methanol and stained with crystal violet staining solution.

### Statistical analysis

All experimental data are shown as the mean (±SD) of at least three independent experiments. Means between two groups were compared by GraphPad Prism 8 using Student *t*-test (two-tailed) or Mann–Whitney U-test. The clinical data were statistically analyzed using SPSS version 23.0. Survival rates were determined by the log-rank (Mantel-Cox) test and compared in Kaplan-Meier plots. *p* < 0.05 was considered statistically significant.

## Supplementary information

Supplemental Fig.1

Supplemental Fig.2

Supplemental Fig.3

supplementary figure legends

Supplementary tables

Supplementary File

original data file of western blots

Reproducibility Checklist

## Data Availability

All data generated or analyzed during this study are included in this published article.

## References

[1] Sung H, Ferlay J, Siegel RL, Laversanne M, Soerjomataram I, Jemal A (2021). Global Cancer Statistics 2020: GLOBOCAN Estimates of Incidence and Mortality Worldwide for 36 Cancers in 185 Countries. CA: A Cancer J Clinicians.

[2] Smyth EC, Nilsson M, Grabsch HI, van Grieken NC, Lordick F (2020). Gastric cancer. Lancet (Lond, Engl).

[3] Song Z, Wu Y, Yang J, Yang D, Fang X (2017). Progress in the treatment of advanced gastric cancer. Tumour Biol: J Int Soc Oncodev Biol Med.

[4] Mohammad HP, Barbash O, Creasy CL (2019). Targeting epigenetic modifications in cancer therapy: erasing the roadmap to cancer. Nat Med.

[5] Yamane K, Tateishi K, Klose RJ, Fang J, Fabrizio LA, Erdjument-Bromage H (2007). PLU-1 is an H3K4 demethylase involved in transcriptional repression and breast cancer cell proliferation. Mol cell.

[6] Swigut T, Wysocka J (2007). H3K27 demethylases, at long last. Cell.

[7] Salminen A, Kaarniranta K, Hiltunen M, Kauppinen A (2014). Histone demethylase Jumonji D3 (JMJD3/KDM6B) at the nexus of epigenetic regulation of inflammation and the aging process. J Mol Med (Berl, Ger).

[8] De Santa F, Totaro MG, Prosperini E, Notarbartolo S, Testa G, Natoli G (2007). The histone H3 lysine-27 demethylase Jmjd3 links inflammation to inhibition of polycomb-mediated gene silencing. Cell.

[9] Lee HY, Choi K, Oh H, Park YK, Park H (2014). HIF-1-dependent induction of Jumonji domain-containing protein (JMJD) 3 under hypoxic conditions. Molecules cells.

[10] Lee HT, Kim SK, Kim SH, Kim K, Lim CH, Park J (2014). Transcription-related element gene expression pattern differs between microglia and macrophages during inflammation. Inflamm Res: Off J Eur Histamine Res Soc..

[11] Arcipowski KM, Martinez CA, Ntziachristos P (2016). Histone demethylases in physiology and cancer: a tale of two enzymes, JMJD3 and UTX. Curr Opin Genet Dev.

[12] Ishii M, Wen H, Corsa CA, Liu T, Coelho AL, Allen RM (2009). Epigenetic regulation of the alternatively activated macrophage phenotype. Blood.

[13] Shan J, Fu L, Balasubramanian MN, Anthony T, Kilberg MS (2012). ATF4-dependent regulation of the JMJD3 gene during amino acid deprivation can be rescued in Atf4-deficient cells by inhibition of deacetylation. J Biol Chem.

[14] Pereira F, Barbáchano A, Silva J, Bonilla F, Campbell MJ, Muñoz A (2011). KDM6B/JMJD3 histone demethylase is induced by vitamin D and modulates its effects in colon cancer cells. Hum Mol Genet.

[15] Burchfield JS, Li Q, Wang HY, Wang RF (2015). JMJD3 as an epigenetic regulator in development and disease. Int J Biochem Cell Biol.

[16] Xun J, Gao R, Wang B, Li Y, Ma Y, Guan J (2021). Histone demethylase KDM6B inhibits breast cancer metastasis by regulating Wnt/β-catenin signaling. FEBS open bio.

[17] Liang S, Yao Q, Wei D, Liu M, Geng F, Wang Q (2019). KDM6B promotes ovarian cancer cell migration and invasion by induced transforming growth factor-β1 expression. J Cell Biochem.

[18] Jiang Y, Li F, Gao B, Ma M, Chen M, Wu Y (2021). KDM6B-mediated histone demethylation of LDHA promotes lung metastasis of osteosarcoma. Theranostics.

[19] Cao Z, Shi X, Tian F, Fang Y, Wu JB, Mrdenovic S (2021). KDM6B is an androgen regulated gene and plays oncogenic roles by demethylating H3K27me3 at cyclin D1 promoter in prostate cancer. Cell Death Dis.

[20] Tang B, Qi G, Tang F, Yuan S, Wang Z, Liang X (2016). Aberrant JMJD3 Expression Upregulates Slug to Promote Migration, Invasion, and Stem Cell-Like Behaviors in Hepatocellular Carcinoma. Cancer Res.

[21] Li Q, Hou L, Ding G, Li Y, Wang J, Qian B (2015). KDM6B induces epithelial-mesenchymal transition and enhances clear cell renal cell carcinoma metastasis through the activation of SLUG. Int J Clin Exp Pathol.

[22] Yang L, Zha Y, Ding J, Ye B, Liu M, Yan C (2019). Histone demethylase KDM6B has an anti-tumorigenic function in neuroblastoma by promoting differentiation. Oncogenesis.

[23] Al Labban D, Jo SH, Ostano P, Saglietti C, Bongiovanni M, Panizzon R (2018). Notch-effector CSL promotes squamous cell carcinoma by repressing histone demethylase KDM6B. J Clin Investig.

[24] He XW, Yu X, Liu T, Yu SY, Chen DJ (2008). Vector-based RNA interference against vascular endothelial growth factor-C inhibits tumor lymphangiogenesis and growth of colorectal cancer in vivo in mice. Chin Med J.

[25] He XW, Liu T, Chen YX, Cheng DJ, Li XR, Xiao Y (2008). Calcium carbonate nanoparticle delivering vascular endothelial growth factor-C siRNA effectively inhibits lymphangiogenesis and growth of gastric cancer in vivo. Cancer gene Ther.

[26] Lee TH, Seng S, Sekine M, Hinton C, Fu Y, Avraham HK (2007). Vascular endothelial growth factor mediates intracrine survival in human breast carcinoma cells through internally expressed VEGFR1/FLT1. PLoS Med.

[27] Gullo I, Grillo F, Mastracci L, Vanoli A, Carneiro F, Saragoni L (2020). Precancerous lesions of the stomach, gastric cancer and hereditary gastric cancer syndromes. Pathologica.

[28] Urosevic J, Blasco MT, Llorente A, Bellmunt A, Berenguer-Llergo A, Guiu M (2020). ERK1/2 Signaling Induces Upregulation of ANGPT2 and CXCR4 to Mediate Liver Metastasis in Colon Cancer. Cancer Res.

[29] Yang J, Zhang L, Jiang Z, Ge C, Zhao F, Jiang J (2019). TCF12 promotes the tumorigenesis and metastasis of hepatocellular carcinoma via upregulation of CXCR4 expression. Theranostics.

[30] Xiao J, Lai H, Wei SH, Ye ZS, Gong FS, Chen L (2019). C. lncRNA HOTAIR promotes gastric cancer proliferation and metastasis via targeting miR-126 to active CXCR4 and RhoA signaling pathway. Cancer Med.

[31] Xue S, Ma M, Bei S, Li F, Wu C, Li H (2021). Identification and Validation of the Immune Regulator CXCR4 as a Novel Promising Target for Gastric Cancer. Front Immunol.

[32] Azar M, Aghazadeh H, Mohammed HN, Sara MRS, Hosseini A, Shomali N (2021). miR-193a-5p as a promising therapeutic candidate in colorectal cancer by reducing 5-FU and Oxaliplatin chemoresistance by targeting CXCR4. Int Immunopharmacol.

[33] Domanska UM, Kruizinga RC, Nagengast WB, Timmer-Bosscha H, Huls G, de Vries EG (2013). A review on CXCR4/CXCL12 axis in oncology: no place to hide. Eur J cancer (Oxf, Engl: 1990).

[34] Mo W, Chen J, Patel A, Zhang L, Chau V, Li Y (2013). CXCR4/CXCL12 mediate autocrine cell- cycle progression in NF1-associated malignant peripheral nerve sheath tumors. Cell.

[35] Zhang W, Fu Z, Yin H, Han Q, Fan W, Wang F (2021). Macrophage Polarization Modulated by Porcine Circovirus Type 2 Facilitates Bacterial Coinfection. Front Immunol.

[36] Li Y, Wang HC, Wang JS, Sun B, Li LP (2020). Chemokine receptor 4 expression is correlated with the occurrence and prognosis of gastric cancer. FEBS open Bio.

[37] Chen G, Zhou Z, Jin J, Zhou Y, Liu Y, Wang W (2021). CXCR4 is a prognostic marker that inhibits the invasion and migration of gastric cancer by regulating VEGF expression. Oncol Lett.

[38] Pajuelo-Lozano N, Alcalá S, Sainz B, Perona R, Sanchez-Perez I (2020). Targeting MAD2 modulates stemness and tumorigenesis in human Gastric Cancer cell lines. Theranostics.

[39] Lin XL, Xu Q, Tang L, Sun L, Han T, Wang LW (2017). Regorafenib inhibited gastric cancer cells growth and invasion via CXCR4 activated Wnt pathway. PloS One.

[40] Dong XZ, Zhao ZR, Hu Y, Lu YP, Liu P, Zhang L (2020). LncRNA COL1A1-014 is involved in the progression of gastric cancer via regulating CXCL12-CXCR4 axis. Gastric Cancer: Off J Int Gastric Cancer Assoc Jpn Gastric Cancer Assoc.

[41] Xiang Z, Zhou ZJ, Xia GK, Zhang XH, Wei ZW, Zhu JT (2017). A positive crosstalk between CXCR4 and CXCR2 promotes gastric cancer metastasis. Oncogene.

[42] D’Errico M, de Rinaldis E, Blasi MF, Viti V, Falchetti M, Calcagnile A (2009). Genome-wide expression profile of sporadic gastric cancers with microsatellite instability. Eur J cancer (Oxf, Engl: 1990).

[43] Ooi CH, Ivanova T, Wu J, Lee M, Tan IB, Tao J (2009). Oncogenic pathway combinations predict clinical prognosis in gastric cancer. PLoS Genet.

[44] Szász AM, Lánczky A, Nagy Á, Förster S, Hark K, Green JE (2016). Cross-validation of survival associated biomarkers in gastric cancer using transcriptomic data of 1,065 patients. Oncotarget.

