# Supplementary figures and images for: KDM6B promotes gastric carcinogenesis and metastasis via upregulation of CXCR4 expression
Source: Cell Death Dis. 2022 Dec 23;13(12):1068. doi: 10.1038/s41419-022-05458-5 (PMC9789124; doi:10.1038/s41419-022-05458-5)

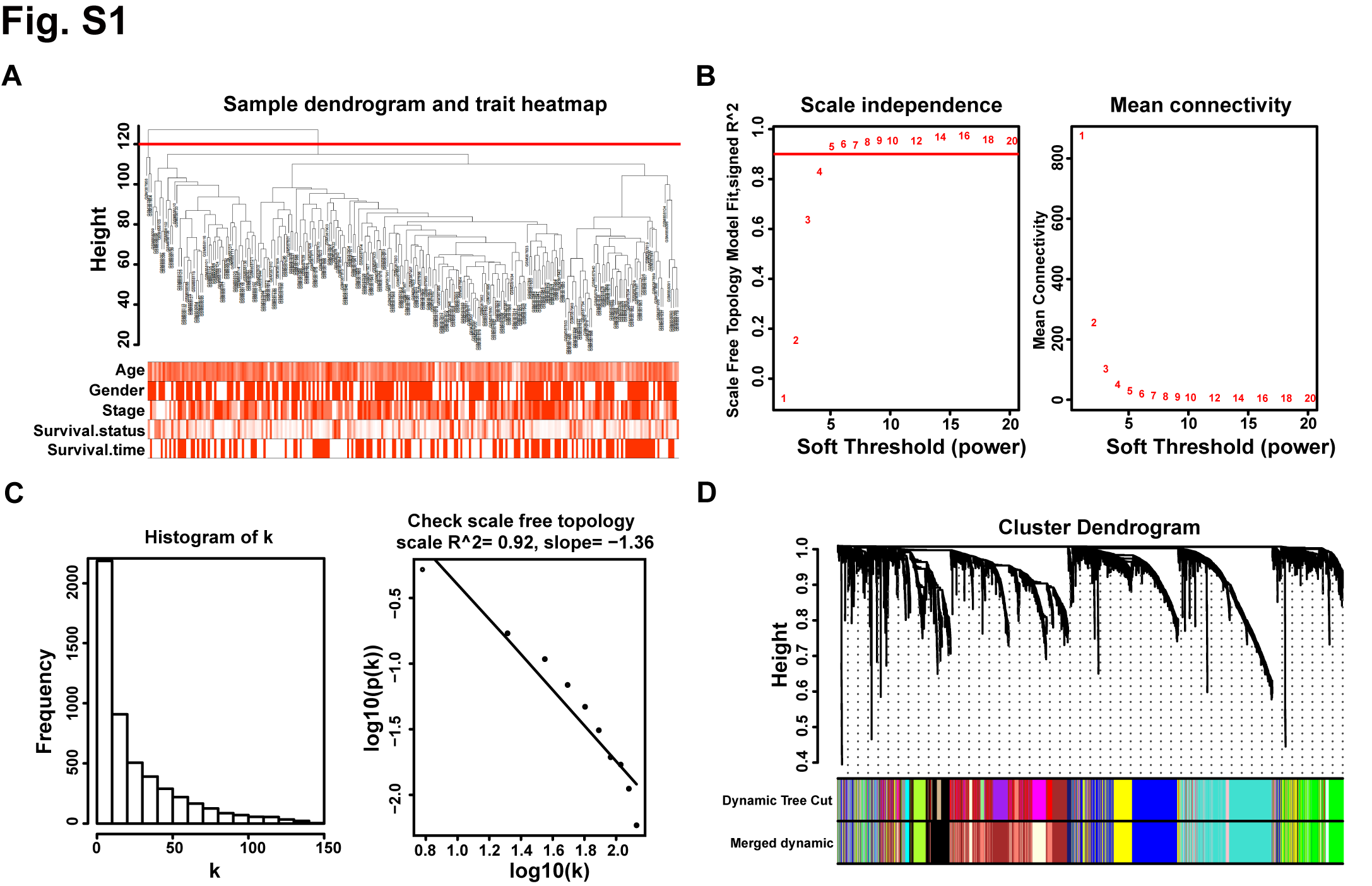

Supplement: Supplementary file 1 — Supplemental Fig.1 [file 41419_2022_5458_MOESM1_ESM.tif]

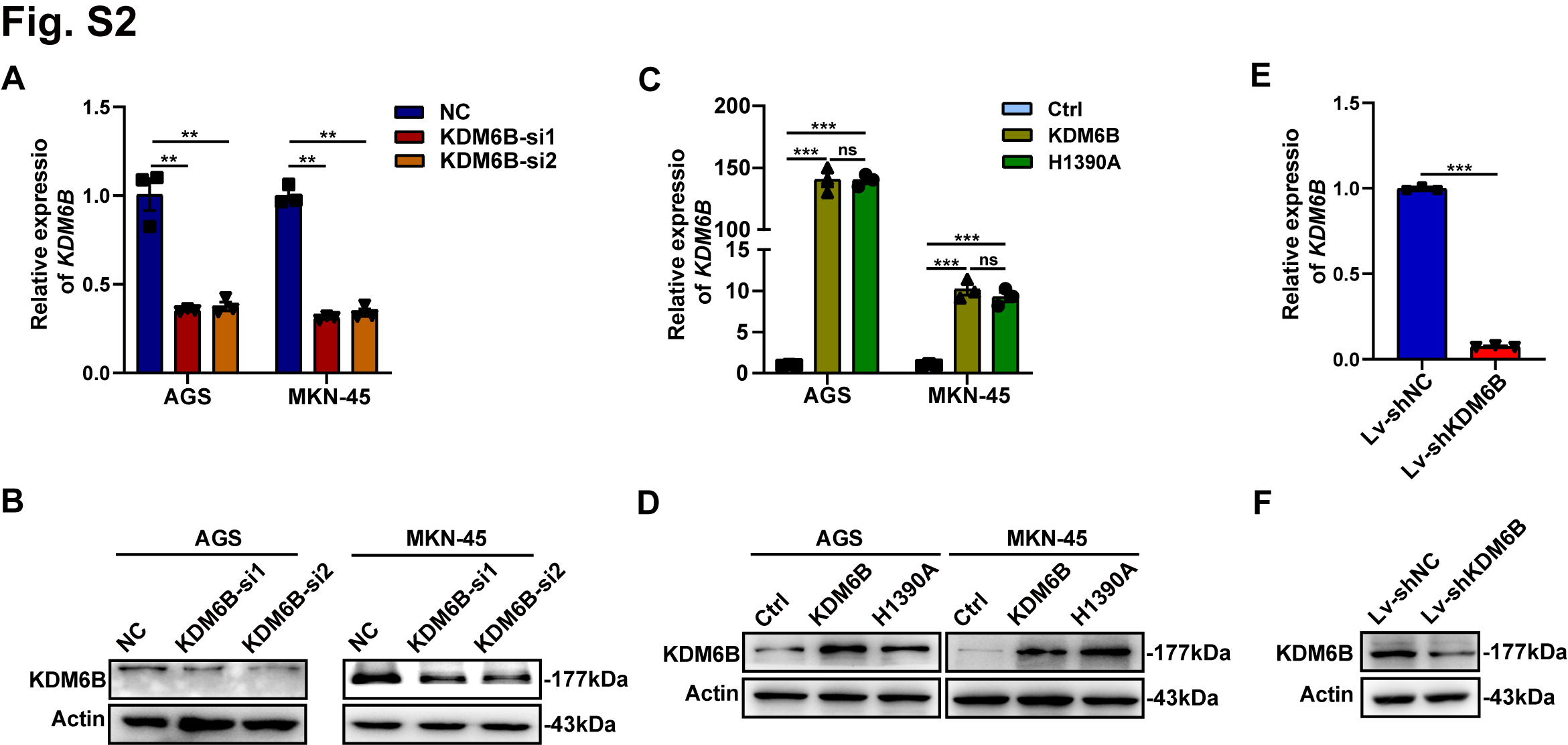

Supplement: Supplementary file 2 — Supplemental Fig.2 [file 41419_2022_5458_MOESM2_ESM.tif]

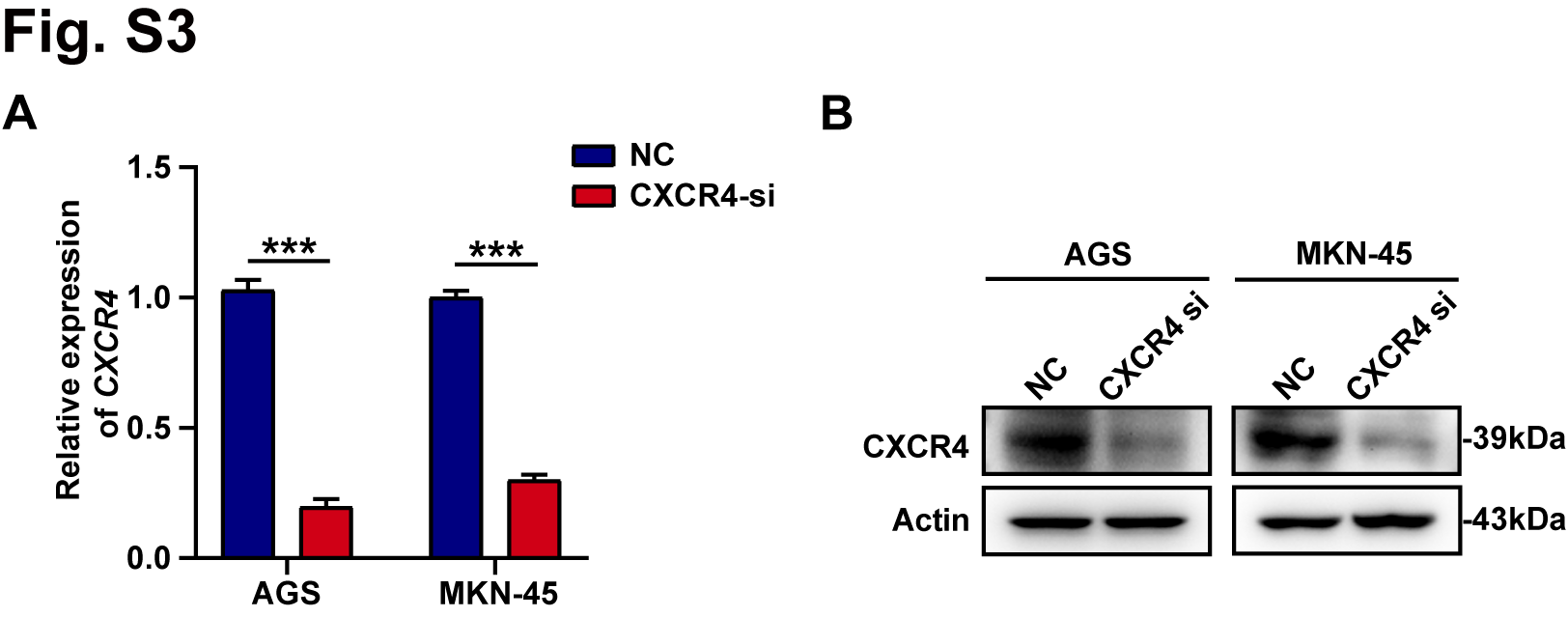

Supplement: Supplementary file 3 — Supplemental Fig.3 [file 41419_2022_5458_MOESM3_ESM.tif]
