# Supplementary material for: KDM6B promotes gastric carcinogenesis and metastasis via upregulation of CXCR4 expression
Source: Cell Death Dis. 2022 Dec 23;13(12):1068. doi: 10.1038/s41419-022-05458-5 (PMC9789124; doi:10.1038/s41419-022-05458-5)
Supplement: Supplementary file 4 — supplementary figure legends [file 41419_2022_5458_MOESM4_ESM.docx]

Supplementary Figure Legends

Supplementary Fig. 1 In the open dataset of gastric cancer, the WGCNA method was used to explore the possible biological functions of KDM6B.

A Cluster tree diagram and heat map of clinical characteristics for the GSE15460 dataset sample, and the samples are clustered based on transcriptomic data, and the cutoff value is set to 120, which is used to reject outlier samples, the bottom red and white graphs show the corresponding clinical characteristics of each sample. B The soft thresholds used to construct the scale-free network are filtered, and the left and right plots show the scale-free fit index (Y-axis) and the average connectivity (Y-axis) as a function of the soft threshold parameters (X-axis), respectively. C The left and right plots are the histogram of the connectivity distribution and the scale-free topological distribution when the soft threshold β is equal to 5, respectively. D The hierarchical clustering tree of genes. The color behavior below the tree chart is different modules after merging, and the different colors represent different gene modules, the genes of the same color module have similar expression profiles.

Supplementary Fig. 2 Small interfering/plasmid transient transfection and the construction of lentiviral stable transfection cell lines. A, B After transfection of KDM6B small interfering siRNA-1, siRNA-2 in two gastric cancer cells, the mRNA (A) and protein (B) expression of KDM6B were detected. C, D After transfection of KDM6B overexpression plasmid (WT or H130A) in two gastric cancer cells, the expression of KDM6B mRNA (C) and protein (D) was detected. E, F The expression of KDM6B mRNA (E) and protein (F) after lentivirus infection of MKN-45 cells. Data are presented as the mean ± SD. n = 3. The *p*-values in Supplementary Fig. 2A, C were calculated by one-way ANOVA. The *p*-value in Supplementary Fig. 2E was calculated by t-test. ns: not significant, ***p* < 0.01, ****p* < 0.001.

Supplementary Fig. 3 Detection of interference efficiency of CXCR4 siRNA in two gastric cancer cell lines. **A, B** After CXCR4 siRNA was transferred into gastric cancer cell lines AGS and MKN-45, the mRNA (A) and protein (B) expression of CXCR4 were detected by RT-qPCR and Western blot. Data are presented as the mean ± SD. n = 3. The *p*-values in Supplementary Fig. 3A were calculated by t-test. ****p* < 0.001.
