# Supplementary material for: KDM6B promotes gastric carcinogenesis and metastasis via upregulation of CXCR4 expression
Source: Cell Death Dis. 2022 Dec 23;13(12):1068. doi: 10.1038/s41419-022-05458-5 (PMC9789124; doi:10.1038/s41419-022-05458-5)
Supplement: Supplementary file 5 — Supplementary tables [file 41419_2022_5458_MOESM5_ESM.docx]

Supplementary Tables S1. siRNAs and shRNAs used in this study

| Genes | Interference sequences |
| --- | --- |
| KDM6B | 5′-CGAAGAACCAUCACAUCAUdTdT-3′（siRNA1） |
|  | 5′-CUGAAGAUCCGGCUCAUCAdTdT-3′（siRNA2） |
| CXCR4 | 5′-GAGGCAGATGACAGATATAdTdT-3′ |
| NC | 5'-AAACGTGACACGTTCGGAGAA-3' |
| KDM6B | 5′-CGAAGAACCAUCACAUCAUdTdT-3′（shRNA） |

Supplementary Tables S2. Quantitative PCR primers used in this study

| Genes | Primer sequences |
| --- | --- |
| KDM6B | Forward: 5′-CGCTGCCTCACCCATATCC-3′ |
|  | Reverse: 5′-ATCCGCGACCTCTGAACTCT-3′ |
| CXCR4 | Forward: 5′-ACTACACCGAGGAAATGGGCT-3′ |
|  | Reverse: 5′-CCCACAATGCCAGTTAAGAAGA-3′ |
| β-Actin | Forward: 5′-AGTTGCGTTACACCCTTTCTTG-3′ |
|  | Reverse: 5′-CACCTTCACCGTTCCAGTTTT-3′ |
| β2-M | Forward: 5′-GAATTGCTATGTGTCTGGGT -3′ |
|  | Reverse: 5′-CATCTTCAAACCTCCATGATG -3′ |

Supplementary Tables S4. Primers used in ChIP assays

| Genes | Primer sequences |
| --- | --- |
| Site A | Forward: 5′-ATCAGTCTCCAGAATTATGCCAAA-3′ |
|  | Reverse: 5′-GCAGCCCATTCAGGAGGTAAAA-3′ |
| Site B | Forward: 5′-AGTTCGAGAGTTTGGGGTCG-3′ |
|  | Reverse: 5′-CTATCCCCGGAGCGCAAAT-3′ |
| Site C | Forward: 5′-GGGAAACCGTTTGGCTCTCT-3′ |
|  | Reverse: 5′-CGCGAGCGTCTTTGAATTGC-3′ |
