# Supplementary material for: KDM6B promotes gastric carcinogenesis and metastasis via upregulation of CXCR4 expression
Source: Cell Death Dis. 2022 Dec 23;13(12):1068. doi: 10.1038/s41419-022-05458-5 (PMC9789124; doi:10.1038/s41419-022-05458-5)

Fig. 1F

KDM6B (#1)

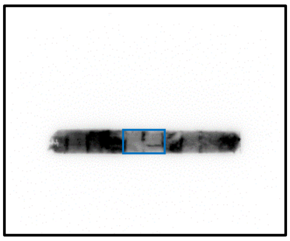

Actin (#1)

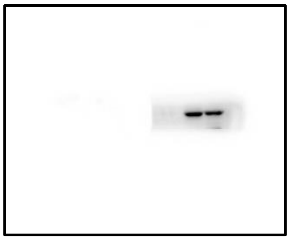

KDM6B (#2)

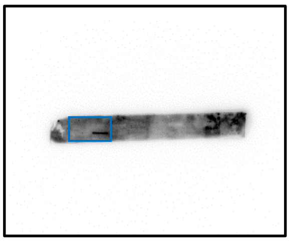

Actin (#2)

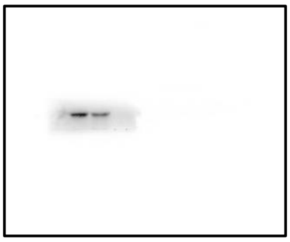

KDM6B (#3)

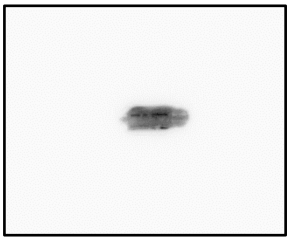

Actin (#3)

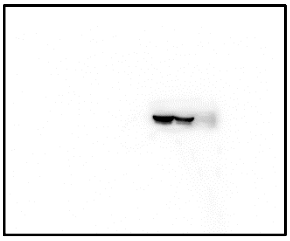

KDM6B (#4)

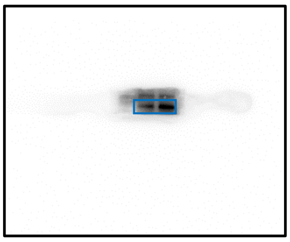

Actin (#3)

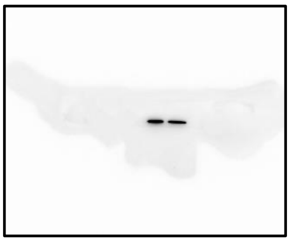

KDM6B (#5)

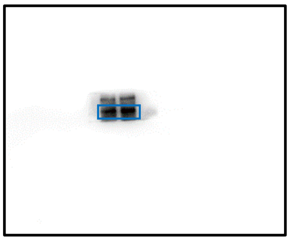

Actin (#5)

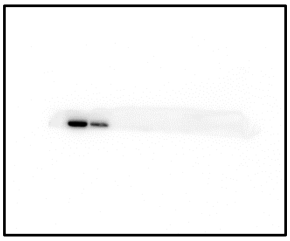

Fig. 3I

KDM6B (AGS)

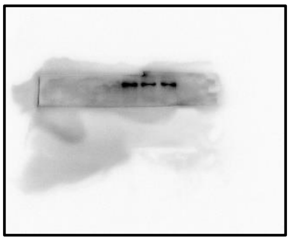

KDM6B (MKN-45)

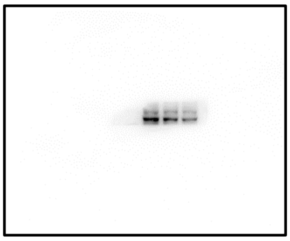

N-Cad (AGS)

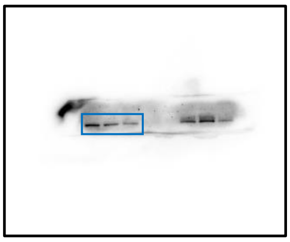

N-Cad (MKN-45)

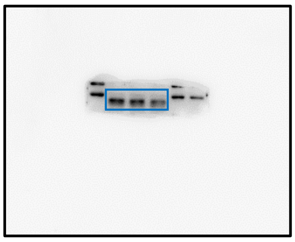

E-Cad (AGS)

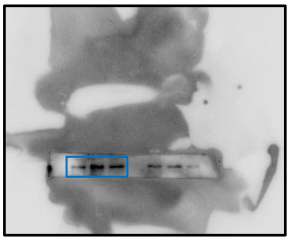

E-Cad (MKN-45)

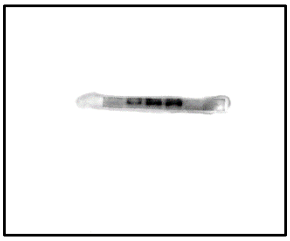

Actin (AGS)

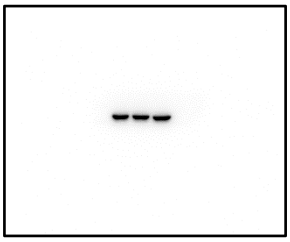

Actin (MKN-45)

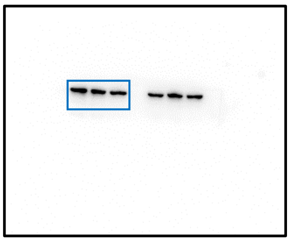

Fig. 3J

KDM6B (AGS)

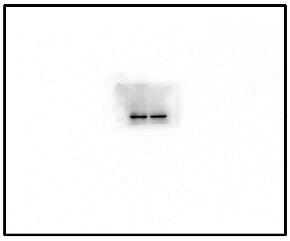

KDM6B (MKN-45)

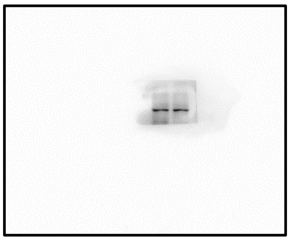

N-Cad (AGS)

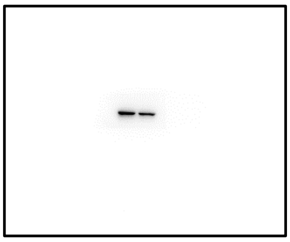

N-Cad (MKN-45)

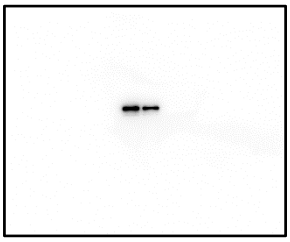

E-Cad (AGS)

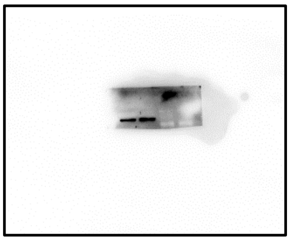

E-Cad (MKN-45)

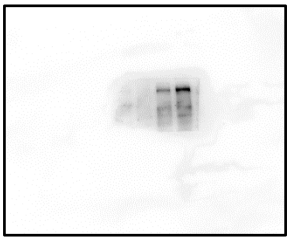

Actin (AGS)

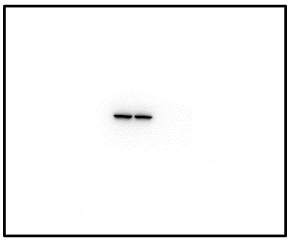

Actin (MKN-45)

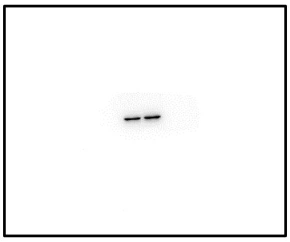

Fig. 3K

KDM6B (AGS)

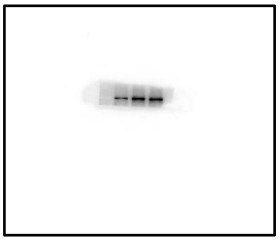

N-Cad (AGS)

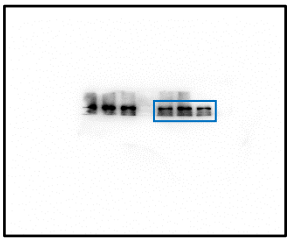

E-Cad (AGS)

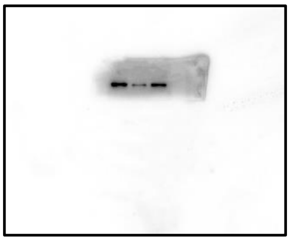

Actin (AGS)

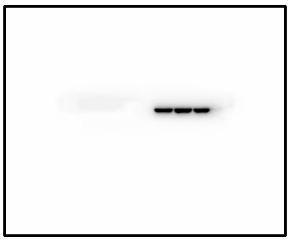

KDM6B (MKN-45)

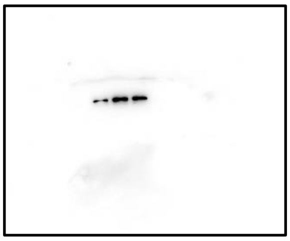

N-Cad (MKN-45)

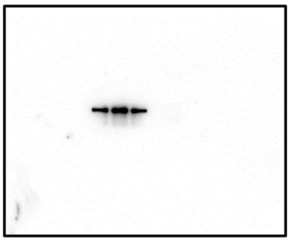

E-Cad (MKN-45)

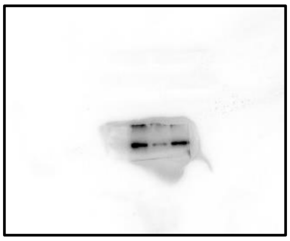

Actin (MKN-45)

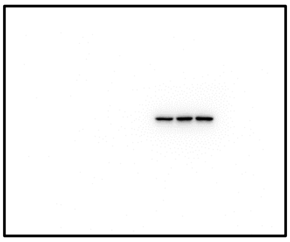

Fig. 4F

CXCR4 (AGS)

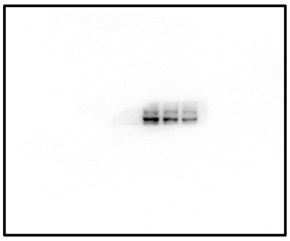

CXCR4 (MKN-45)

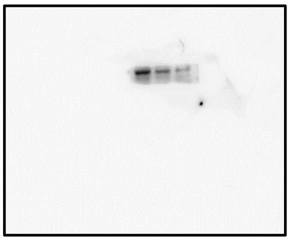

p-ERK (AGS)

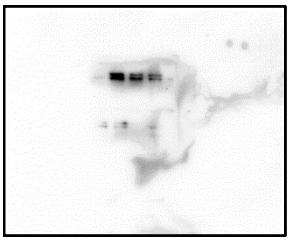

p-ERK (MKN-45)

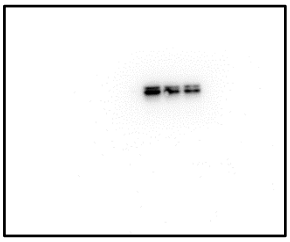

ERK (AGS)

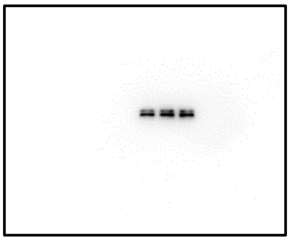

ERK (MKN-45)

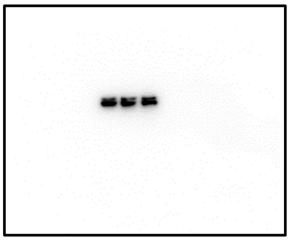

p-AKT (AGS)

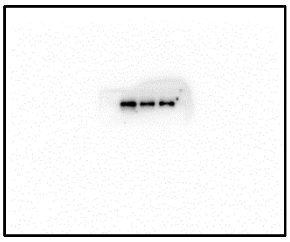

p-AKT (MKN-45)

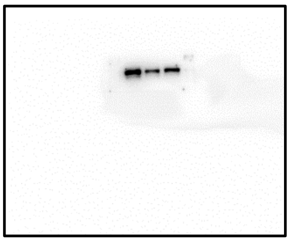

AKT (AGS)

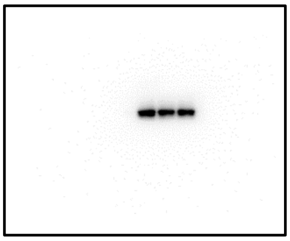

AKT (MKN-45)

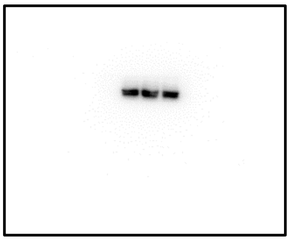

Actin (AGS)

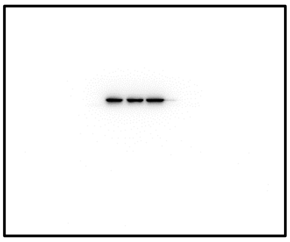

Actin (MKN-45)

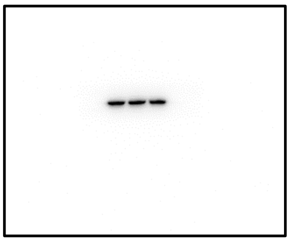

Fig. 4H

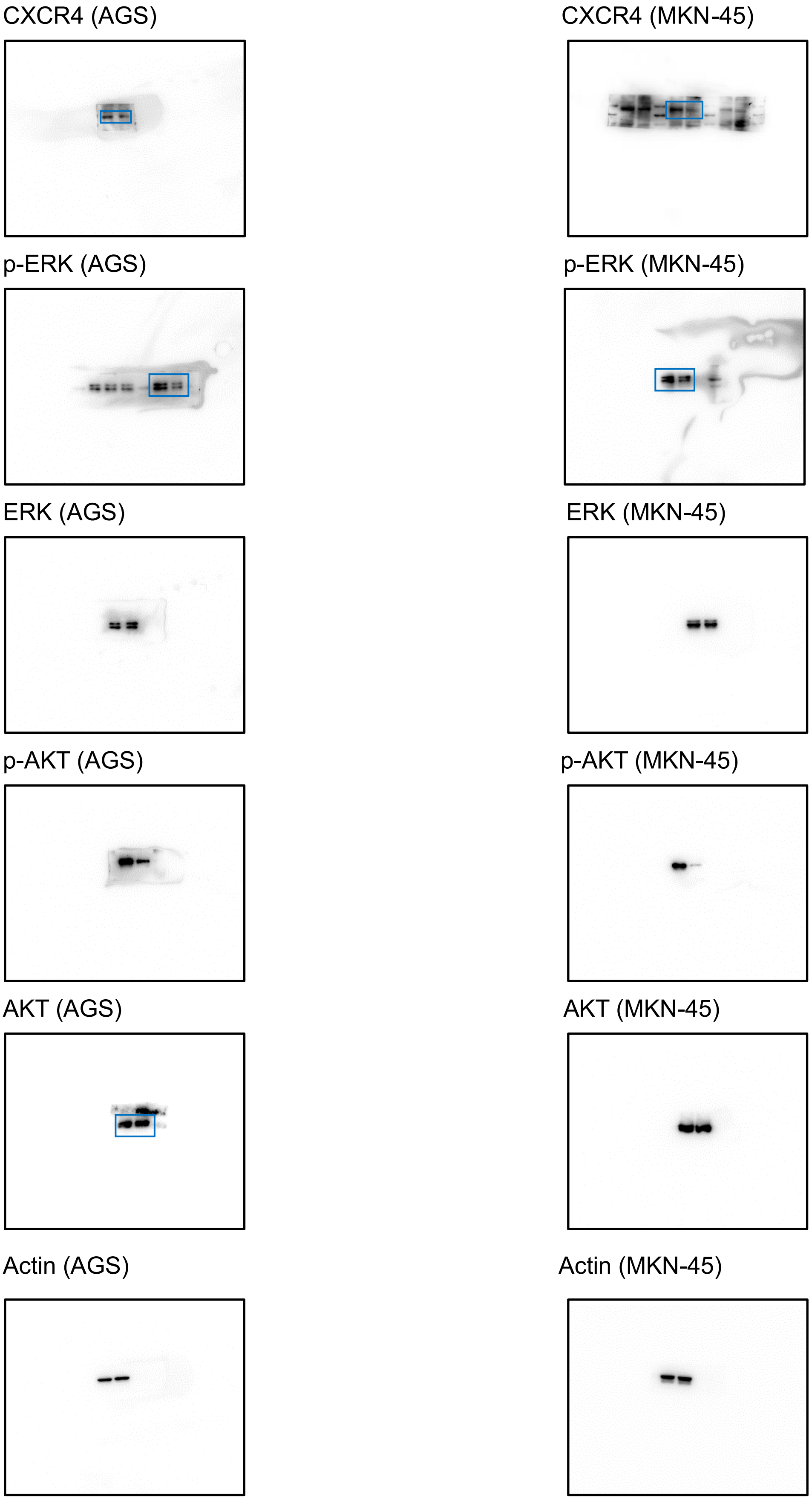
CXCR4 (AGS)
CXCR4 (MKN-45)
p-ERK (AGS)
p-ERK (MKN-45)
ERK (AGS)
ERK (MKN-45)
p-AKT (AGS)
p-AKT (MKN-45)
AKT (AGS)
AKT (MKN-45)
Actin (AGS)
Actin (MKN-45)

Fig. 4J

CXCR4 (AGS)

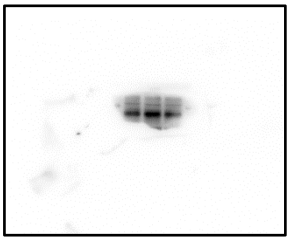

CXCR4 (MKN-45

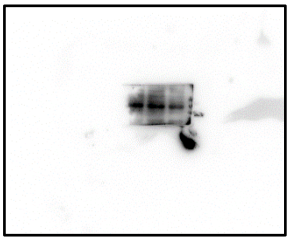

p-ERK (AGS)

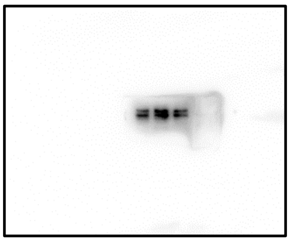

p-ERK (MKN-45)

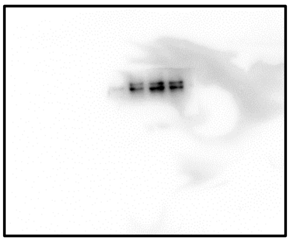

ERK (AGS)

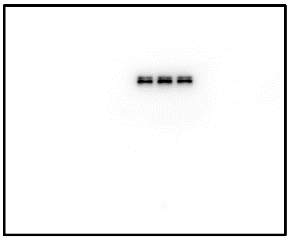

ERK (MKN-45)

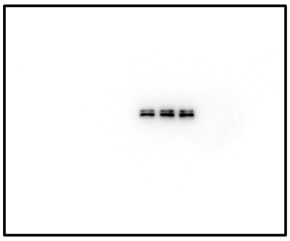

p-AKT (AGS)

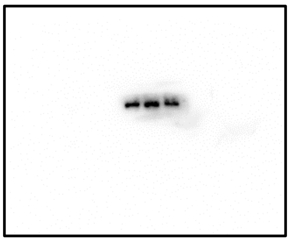

p-AKT (MKN-45)

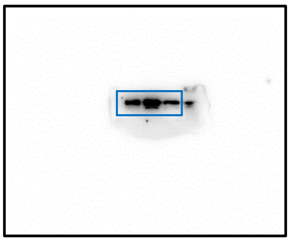

AKT (AGS)

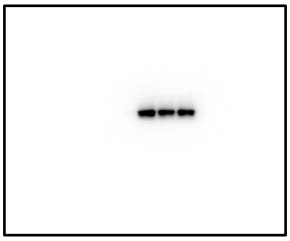

AKT (MKN-45)

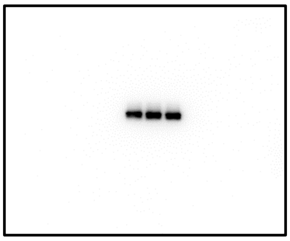

Actin (AGS)

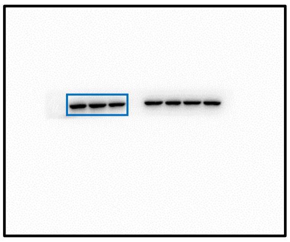

Actin (MKN-45)

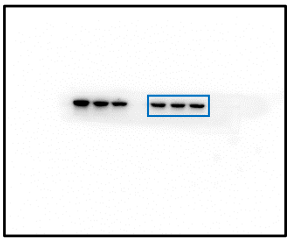

Fig. 5A

H3K27me3 (AGS)

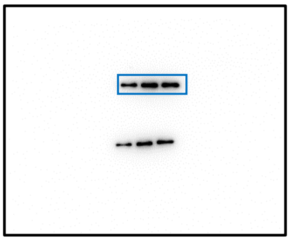

H3K27me3 (MKN-45)

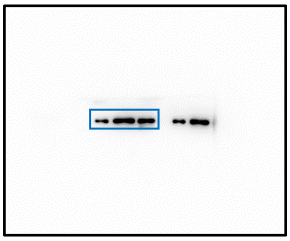

H3 (AGS)

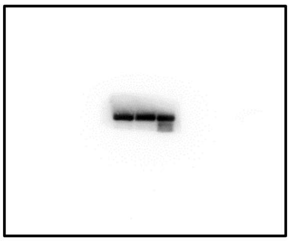

H3 (MKN-45)

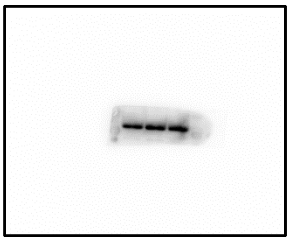

Fig. 5B

H3K27me3 (AGS)

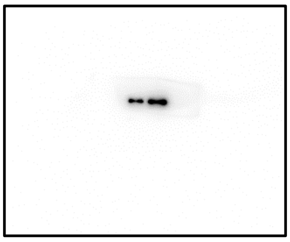

H3K27me3 (MKN-45)

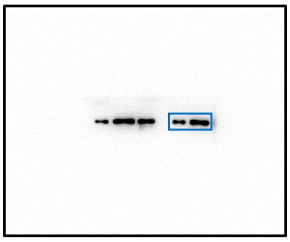

H3 (AGS)

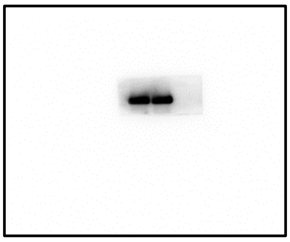

H3 (MKN-45)

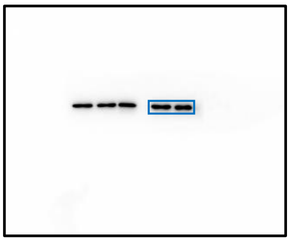

Fig. 7B

CagA (AGS)

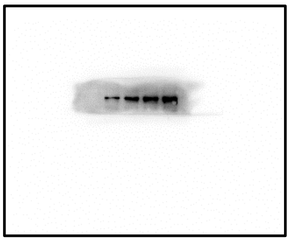

KDM6B (AGS)

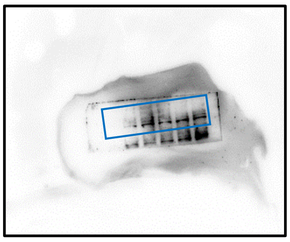

Actin (AGS)

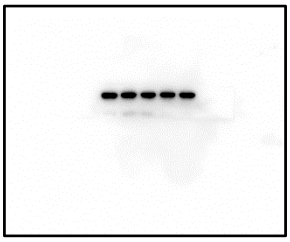

CagA (MKN-45)

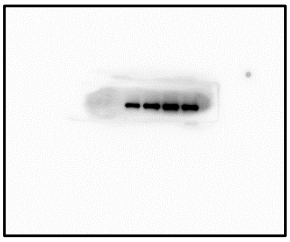

KDM6B (MKN-45)

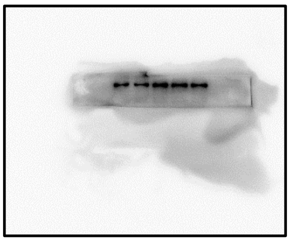

Actin (MKN-45)

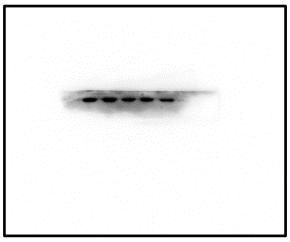

Fig. 7D

CagA (AGS)

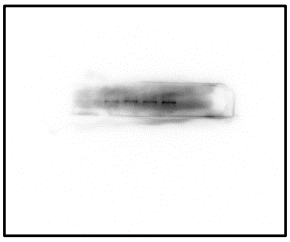

CagA (MKN-45)

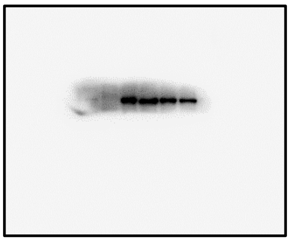

KDM6B (AGS)

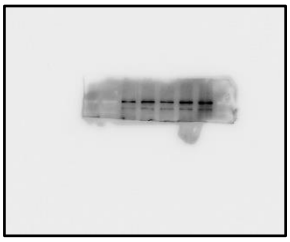

KDM6B (MKN-45)

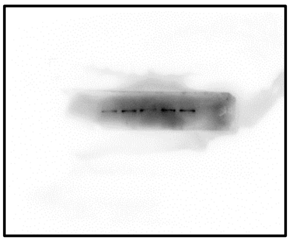

Actin (AGS)

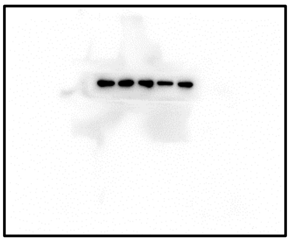

Actin (MKN-45)

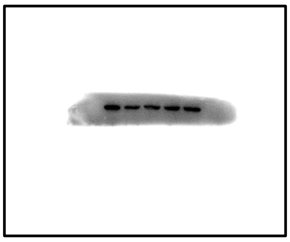

Supplementary Fig. 2B

KDM6B (AGS)

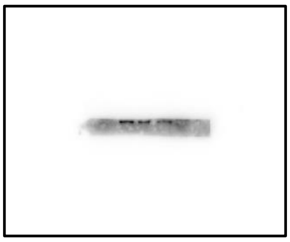

KDM6B (MKN-45)

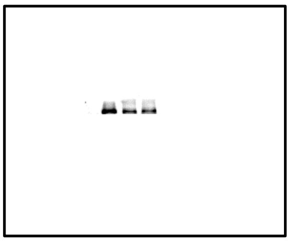

Actin (AGS)

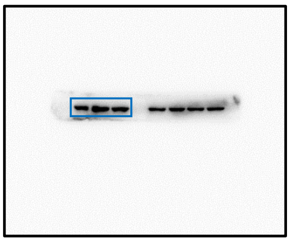

Actin (MKN-45)

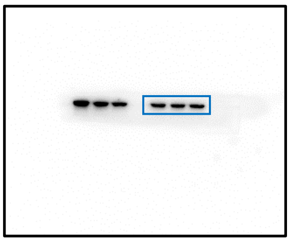

Supplementary Fig. 2D

KDM6B (AGS)

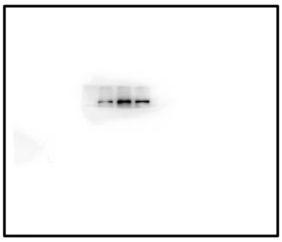

KDM6B (MKN-45)

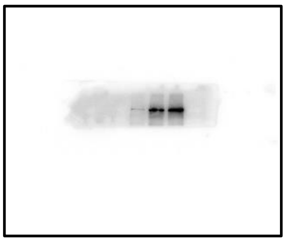

Actin (AGS)

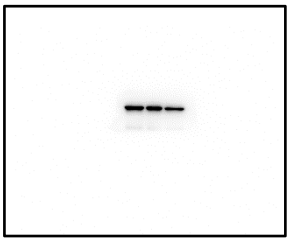

Actin (MKN-45)

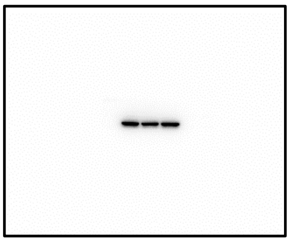

Supplementary Fig. 2F

KDM6B (MKN-45)

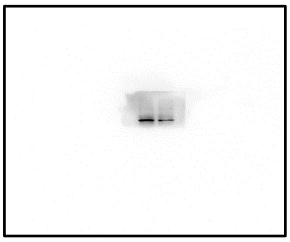

Actin (MKN-45)

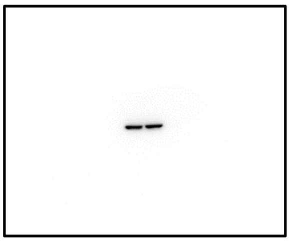

Supplemental Fig. 3B

CXCR4 (AGS)

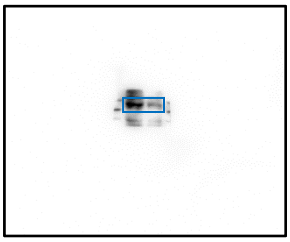

CXCR4 (MKN-45)

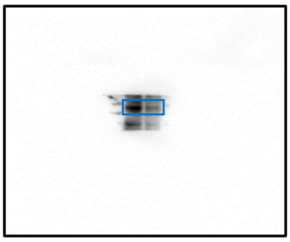

Actin (AGS)

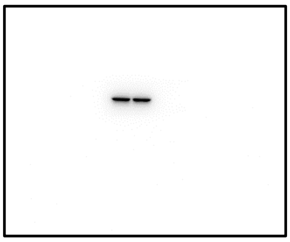

Actin (MKN-45)

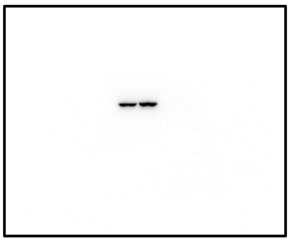

Supplement: Supplementary file 7 — original data file of western blots [file 41419_2022_5458_MOESM7_ESM.pdf]
